# Supplementary figures and images for: Lipopolysaccharide triggers different transcriptional signatures in taurine and indicine cattle macrophages: Reactive oxygen species and potential outcomes to the development of immune response to infections
Source: PLoS One. 2020 Nov 6;15(11):e0241861. doi: 10.1371/journal.pone.0241861 (PMC7647108; doi:10.1371/journal.pone.0241861)

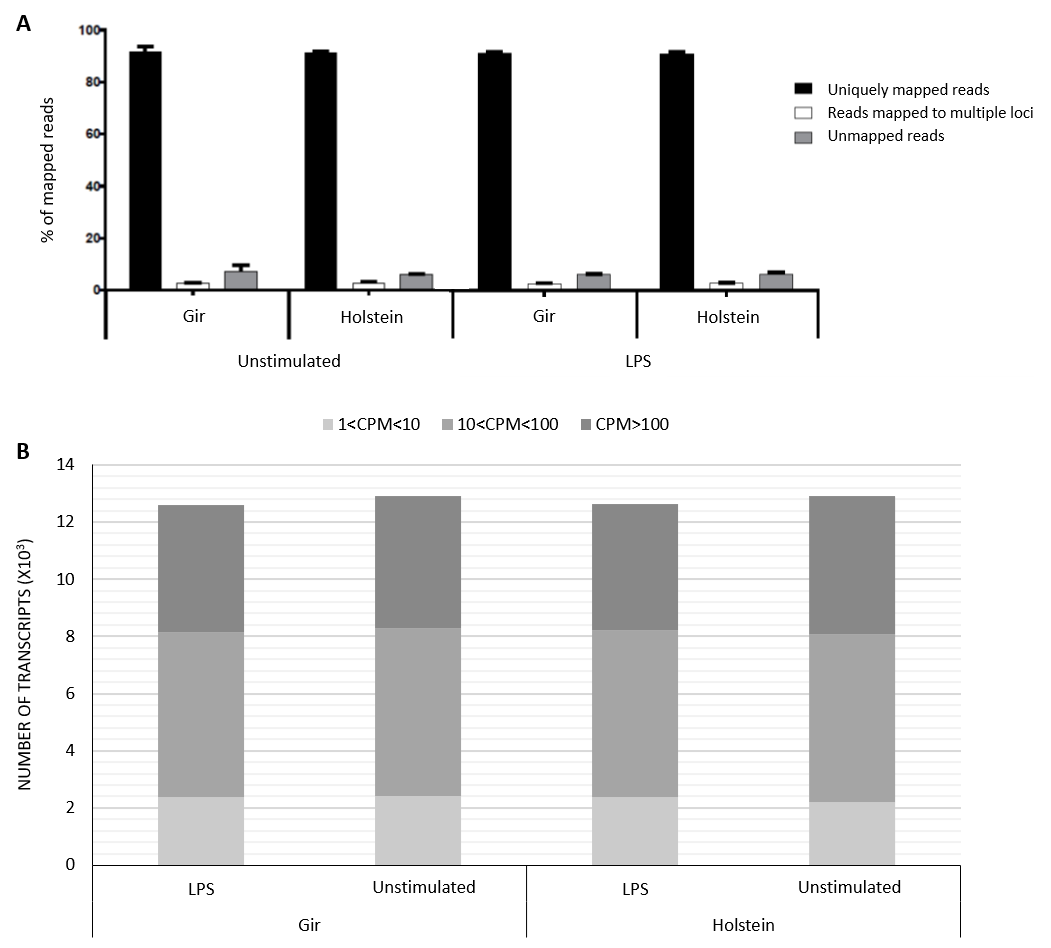

Supplement: S1 Fig — Percentage of reads mapped to ARS-UCD1.2 bovine reference genome. (A) Reads mapped for each breed and treatment (unstimulated and LPS); (B) Total number of transcripts detected and categorized according to average of in vitro gene expression levels (CPM, counts per million) in unstimulated and LPS treated MDMs from Holstein ang Gir breeds. (TIF) [file pone.0241861.s001.tif]

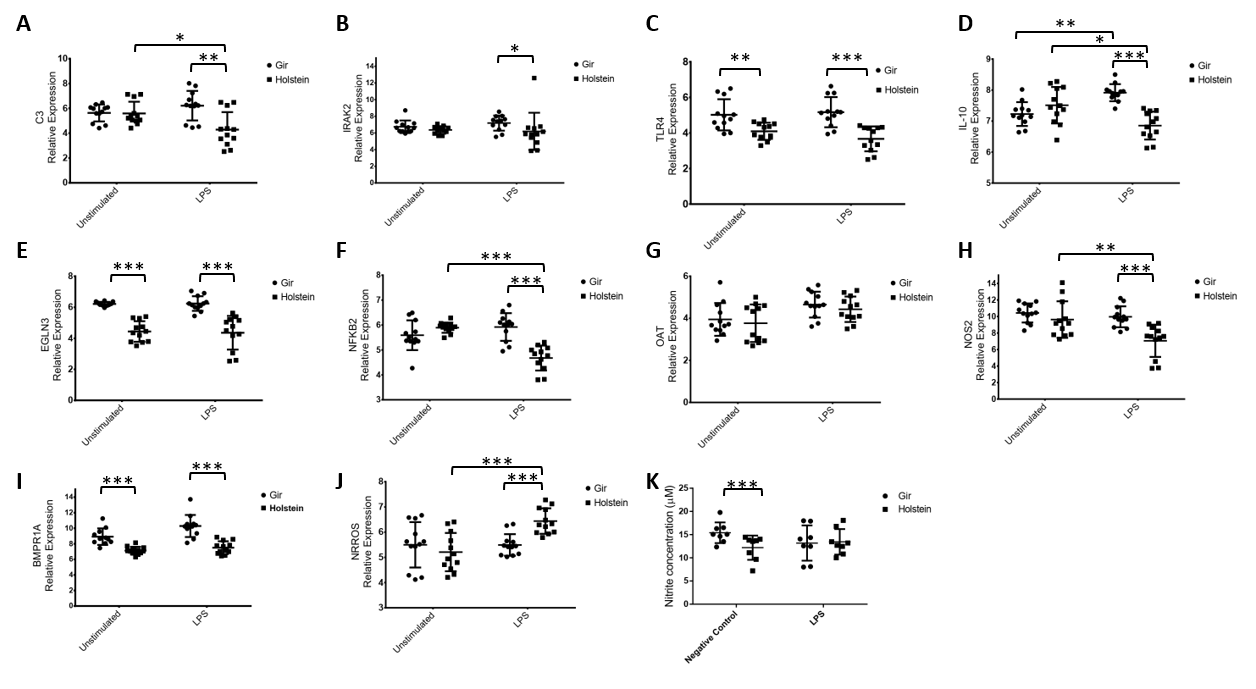

Supplement: S2 Fig — (A-J) RT-qPCR of unstimulated and LPS-treated MDMs from Holstein (n = 4) and Gir (n = 4) bovine breeds. Data shown as average ± SD of three replicates for each animal. T-test was used for comparisons between breeds and one-way analysis of variance between different stimuli into the same breed. *P<0.05, **P<0.01, ***P<0.001. (K) Levels of nitrite at unstimulated and LPS (10ng/ml) treated MDM cell culture supernatant from Holstein and Gir breeds after 48hours of stimulation. Data shown as concentration average ± SD for each group. T-test was used for comparisons between breeds (*P<0.05) and one-way analysis variance between different stimuli within the same breed. (TIF) [file pone.0241861.s002.tif]
