# Supplementary material for: Lipopolysaccharide triggers different transcriptional signatures in taurine and indicine cattle macrophages: Reactive oxygen species and potential outcomes to the development of immune response to infections
Source: PLoS One. 2020 Nov 6;15(11):e0241861. doi: 10.1371/journal.pone.0241861 (PMC7647108; doi:10.1371/journal.pone.0241861)
Supplement: S1 Table — Gene symbol, name and primer sequence of all primers designed for RT-qPCR analyses. RPLP0 and Ubiquitin used as reference genes (lowest values of average expression stability M according to GeNORM). Tm: melting temperature; Fwd: forward primer; Rev: reverse primer. (PDF) [file pone.0241861.s003.pdf]

Gene Symbol	Gene name	Tm (ºC)	Sequence (5'-3')	Average expression stability for housekeeping genes (M)
C3	Complement Factor 3	64.7	Fwd - GCTGCACGAGGCTAAAGACAT	
		65	Rev - TTCGCAACTCTCTGTAGTGGTTTT	-
EGLN3	EGL-9 family hypoxia inducible factor 3	64.1	Fwd - TGCTTTCAGGTTCTTGCTTTGAT	
		63.6	Rev - GGCTACGGGAACACAACAGAGT	-
GATA3	GATA Binding Protein 3	64.2	Fwd - TGCTAAACTACCCCGCAAAGAT	
		62.5	Rev - GGGTCGTTTAAGGATTTGTTTTCA	-
IL10	Interleukin 10	66.2	Fwd - GCACTACTCTGTTGCCTGGTCTT	
		64.8	Rev - GCTGGTTGGCAAGTGGATACA	-
IRAK2	Interleukin-1 receptor-associated kinase-like 2	64.5	Fwd - AACATTGGAGCTCTTGACATTGC	
		63.9	Rev - TCCAGGGCTATCCCACTTTG	-
NFκB2	Nuclear Factor Kappa B Subunit 2	63.8	Fwd - ATCTTGGCAGGTCCCTCGTA	
		64.7	Rev - CCAATGCGTGTGCCTGAA	-
NOS2	Nitric Oxide Synthase 2	64.8	Fwd - GCAGCGGAGTGACTTTCCAA	
		62.9	Rev - GGATGCCAGGCAAGACTTG	-
NRROS	Negative Regulator Of Reactive Oxygen Species	63.2	Fwd - TCATATCCAGGAAGCGGAGACT	
		64.5	Rev - CGAGAAGGGCCGGAAGAC	-
OAT	Ornithine Aminotransferase	64.5	Fwd - CCAACCCACGGCGACAT	
		63.2	Rev - TCTAGAATCTCATCCTCCTTGATCAC	-
TLR4	Toll Like Receptor 4	62.8	Fwd - TTGGTACATGGCGGCATTTA	
		62.4	Rev - CCGAGTGAGCCGGAATGA	-
RPLP0	Ribosomal Protein Lateral Stalk Subunit P0	62.9	Fwd - CAACCCTGAAGTGCTTGACAT	
		63	Rev - AGGCAGATGGATCAGCCA	0.4
Ubiquitin	Ubiquitin	66.3	Fwd - GGCAAGACCATCACCCTGGAA	
		67.5	Rev - GCCACCCCTCAGACGAAGGA	0.338
18S	18S ribosomal RNA	65	Fwd - GTAACCCGTTGCACCCCATT	
		61.8	Rev - CCATCCAATCGGTAGTAGCG	0.473
GAPDH	Glyceraldehyde-3-Phosphate Dehydrogenase	56.8	Fwd - GGCGTGAACCACGAGAAGTATAA	
		57.8	Rev - CCCTCCACGATGCCAAAGT	0.419
