# Supplementary material for: Lipopolysaccharide triggers different transcriptional signatures in taurine and indicine cattle macrophages: Reactive oxygen species and potential outcomes to the development of immune response to infections
Source: PLoS One. 2020 Nov 6;15(11):e0241861. doi: 10.1371/journal.pone.0241861 (PMC7647108; doi:10.1371/journal.pone.0241861)
Supplement: S2 Table — Differential expression was performed on RNA sequencing data from unstimulated and LPS (100ng/ml) treated MDMs from Holstein breed. Genes that showed statistical differences in contrast (LogFC≥1; CPM>1; FDR<0.05) are shown. (PDF) [file pone.0241861.s004.pdf]

Gene Symbol	logFC	FDR
MSR1	-2.21292284	0.0004733
MRC2	-2.398648613	0.0006521
FOS	-1.756857713	0.0009176
XG	-1.965111841	0.0020473
EPB41L3	-1.919615862	0.0022081
VSIG4	-3.457768457	0.002293
PLAC8B	-2.18130999	0.0039939
SORBS3	-2.372288515	0.0050521
BOLA-DMA	-3.539890386	0.0061843
VAT1	-1.530904492	0.0066006
CDH5	-2.868188865	0.0097406
CHN1	-2.770098141	0.0104452
UACA	-1.829792004	0.0104452
CTTNBP2	-2.365946364	0.0116873
SLC16A1	-1.544781199	0.0129748
COL14A1	-1.884365113	0.0149187
NRROS	-1.543223263	0.0163977
PLEKHA4	-2.271745786	0.016752
ACSF2	-1.816788672	0.0197429
PALD1	-3.150101257	0.020175
MGST1	-1.947375279	0.020175
TRIM47	-1.685997974	0.0226179
SLC37A2	-2.576086545	0.0227131
ITGB5	-1.55833423	0.0235698
ASPA	-3.813981075	0.0238186
SAMD11	-2.354295021	0.0255372
BIN1	-1.523909604	0.0268806
ITSN1	-1.375829162	0.0312359
LOC782367	-1.705443073	0.034429
CYP27A1	-3.362313078	0.034547
SLC9A9	-1.573098536	0.0361887
CDC42EP3	-1.445884303	0.0375674
CSPG4	-2.713507908	0.0375674
PPARG	-1.909284298	0.0381846
AXL	-2.23724276	0.0408056
CACNA1G	-2.74977991	0.0411892
AKR1B1	-1.559384816	0.0412412
GSTM1	-1.953954201	0.04358
CHI3L2	4.417354645	9.03E-10
SCNN1D	3.083157799	4.32E-09
MCEMP1	3.096656927	2.72E-08
RAMP3	4.443025321	1.04E-07
EHF	2.845106597	1.10E-07
EBI3	2.81946928	1.10E-07
SH2D4A	3.85559296	4.08E-07
RETN	3.31526366	6.99E-07
CSF3	4.396190612	1.75E-06
IL17REL	2.43949045	1.75E-06
SLC28A3	3.359402222	1.25E-05

LPAR3	2.432350415	1.60E-05
GJB2	2.722052816	3.59E-05
GATA3	3.769463899	5.70E-05
WFDC18	4.760345706	6.38E-05
SOD2	2.488793265	6.38E-05
SAA3	2.690210178	0.0001164
TNIP3	1.935686285	0.0001327
AVIL	4.385677274	0.0001327
ENSBTAG00000052099	2.806655336	0.0001327
CA12	2.036375117	0.000162
IL23R	2.574176294	0.0002036
SOCS1	2.161554959	0.0007382
CXCL3	2.085648679	0.0009938
CCL4	2.907426301	0.0010796
PIM3	1.682974428	0.0010796
ACOD1	2.191547121	0.0014644
LOC104968478	2.627978056	0.0018086
S100A8	2.475254207	0.0018153
BCL2A1	1.64204396	0.0022547
IL1A	2.738822939	0.0033524
LOC784768	2.540144284	0.0066006
NLRP12	2.23961399	0.0068646
SLC13A5	1.503245859	0.0092329
LOC281376	2.340847991	0.0092329
S100A9	2.507183	0.0116932
MARCO	2.532984732	0.014025
RUBCNL	1.948009761	0.0159646
GRO1	1.61072669	0.0159646
CNTFR	1.879075618	0.0164832
ZC3H12A	1.426165698	0.0164832
PDPN	1.865948996	0.016752
VLDLR	1.423717776	0.0197429
NXPE4	3.409499993	0.020175
CLDN1	2.596748129	0.020175
MT2A	1.959747823	0.0226302
IL1B	2.824343701	0.0227131
ABCA3	1.342844859	0.0227131
POU2F2	1.323713477	0.0227131
EDN1	3.744384074	0.0230342
MEFV	1.626555669	0.0230345
KCNJ15	2.627106062	0.0238186
GPR84	2.206527039	0.0268806
B3GNT3	2.231616765	0.0333003
A4GALT	1.566958707	0.0336913
CCL5	1.521988127	0.0340291
GNG5	1.200437508	0.0340291
TGM1	2.121867927	0.035783
CXCL2	2.361699811	0.0373185
ENSBTAG00000048500	1.407384959	0.0395133
ENSBTAG00000002290	1.780183671	0.041688
