# Supplementary material for: Lipopolysaccharide triggers different transcriptional signatures in taurine and indicine cattle macrophages: Reactive oxygen species and potential outcomes to the development of immune response to infections
Source: PLoS One. 2020 Nov 6;15(11):e0241861. doi: 10.1371/journal.pone.0241861 (PMC7647108; doi:10.1371/journal.pone.0241861)
Supplement: S3 Table — Differential expression was performed on RNA sequencing data from unstimulated and LPS (100ng/ml) treated MDMs from Gir breed Genes that showed statistical differences in contrast (LogFC≥1; CPM>1; FDR<0.05) are shown. (PDF) [file pone.0241861.s005.pdf]

Gene Symbol	logFC	FDR
JAML	-3.056259163	9.34E-12
TIMD4	-3.768740131	1.88E-07
MRC2	-2.309150512	3.18E-07
MAOA	-2.04970505	4.81E-07
KANK2	-3.06525994	3.19E-06
LAMB2	-5.354199804	3.47E-06
IFIT3	-2.759423523	4.72E-06
COL3A1	-8.096874654	8.84E-06
AXL	-4.939127517	9.08E-06
SERPINH1	-6.05411609	2.10E-05
TIMP3	-5.98248459	2.10E-05
BOLA-DMB	-2.78277823	2.13E-05
LOC100139670	-2.938408488	2.27E-05
ITGB5	-1.466354012	2.89E-05
MAP1A	-5.235322843	3.39E-05
CCN2	-6.331908398	5.16E-05
CXCR4	-1.525835986	5.67E-05
FN1	-4.85269839	5.67E-05
GPNMB	-2.436525595	5.96E-05
NES	-5.133984938	6.19E-05
SPARC	-6.829067775	7.19E-05
NDRG2	-1.450930225	7.27E-05
CCN1	-5.714978259	7.71E-05
CYP27A1	-2.526880004	7.71E-05
TPM2	-4.183466222	8.87E-05
FBLN2	-7.338892589	0.000104705
PTPRF	-3.58931082	0.000104705
COL1A1	-7.498454581	0.000104705
CAVIN1	-4.950249495	0.000106315
COL1A2	-7.302343301	0.000132844
SLC9A9	-1.139809061	0.000144646
CERCAM	-3.43804166	0.000150613
FSTL1	-5.136375606	0.000169652
LOXL2	-7.018776064	0.00020862
DDAH2	-3.836389535	0.00021599
GPR183	-1.806370406	0.000216722
PSAT1	-1.871550644	0.000223155
CDC42EP3	-1.548499925	0.000237257
GABARAPL1	-1.254045795	0.000307847
OLFML3	-3.94463119	0.0003589
COL6A3	-2.552991665	0.000383403
ALDH1L2	-3.554995111	0.000428635
ACSF2	-1.501548401	0.000428635
ASPA	-2.921382866	0.000429136
CREB3L1	-6.37291242	0.000429136
ASNS	-2.914421199	0.000429136
CD81	-1.189149929	0.000508605
PEF1	-1.216401359	0.000521489
C1QA	-1.258600774	0.000521489

FCER2	-1.989778844	0.000521489
SDC2	-3.025969773	0.000527369
TAGLN	-6.225775248	0.000556697
VSIG4	-1.328926311	0.000556697
TNC	-7.109890318	0.000558254
CXXC5	-2.092585646	0.000605287
QSOX1	-2.82844152	0.000607544
ABI3	-1.789080338	0.000607544
RFTN1	-2.243414677	0.000646069
FBN1	-7.30429187	0.000646069
SERPINE1	-4.571726289	0.000646069
PRSS23	-4.105383239	0.000646069
VDR	-1.36279406	0.000656849
FSCN1	-2.83924746	0.000816944
SLC15A3	-1.342459941	0.00089103
PLEKHA4	-3.106804466	0.000962702
AKR1B1	-1.487108591	0.000978101
ADGRA2	-2.028860183	0.000978101
COL5A2	-6.991522312	0.001019919
AMOTL2	-4.307242418	0.001019919
ROR2	-2.702827036	0.001107085
ACTG2	-3.755131316	0.00112379
ALDH1A1	-1.728765179	0.001219452
COL6A2	-6.740469027	0.001219452
CRISPLD2	-3.559733439	0.001219452
FST	-6.372107636	0.001264251
FEZ1	-3.061929757	0.001275954
ARAF	-2.823191003	0.001275954
SLC8B1	-1.053590357	0.001352085
RAB13	-2.907746142	0.001781523
EPHB3	-1.54630063	0.001804604
MMP2	-6.076405288	0.001986215
GAL3ST4	-2.334689232	0.002025307
PTK7	-3.682853875	0.002060192
VEGFA	-1.201550497	0.002188916
CDH11	-5.166450567	0.002211271
FLNC	-5.709974603	0.002362434
COL6A1	-6.622840216	0.002362635
MYH10	-3.831526267	0.002466641
RGS1	-1.194889552	0.002466641
VAT1	-1.115779043	0.002466641
PTPRS	-3.926932178	0.002513437
PC	-1.076250099	0.002928834
ARHGEF10	-2.404733749	0.002941224
PIK3IP1	-1.550162141	0.003090182
NXN	-2.17755106	0.00365149
ABCB6	-1.054637426	0.003956595
SULF2	-3.757225094	0.003956595
PALLD	-3.123465187	0.004103957
TYRO3	-1.215419284	0.004160065

TGFBI	-1.077333501	0.004365884
COL12A1	-5.972241466	0.004513258
BLNK	-1.961210523	0.004611711
NQO1	-1.262827805	0.004786329
GAS6	-1.532752582	0.004786329
C1QTNF12	-2.288770129	0.005041657
GPR132	-1.505627113	0.005199935
FKBP10	-3.29246925	0.005319944
C1QC	-1.148300713	0.005319944
BOLA-DMA	-2.449206615	0.005499573
CLEC7A	-1.575490724	0.005499573
LOC782367	-1.141503978	0.005499573
MAP1B	-3.515056618	0.005604406
ITGA3	-2.609410196	0.005604406
SLC37A2	-1.157405225	0.005748631
CBR1	-1.578778517	0.005818517
HSPB6	-2.515759783	0.005852915
POSTN	-3.622536527	0.006001803
CTSF	-0.945720222	0.006160446
DAB2	-1.513983226	0.006160446
TREML1	-1.670051196	0.006569646
PCK2	-1.1584285	0.006615592
ALOX5	-2.035546667	0.006655226
CSF1	-3.280153239	0.006885718
NREP	-2.151323473	0.00735887
PALD1	-1.634026336	0.007821639
CRIM1	-2.117958298	0.007857247
KCNT1	-0.908211947	0.007928382
GNG5	0.860401511	0.008809712
CA11	-1.585724952	0.008889786
GPT2	-1.416652538	0.009135868
MFGE8	-2.097668865	0.009160613
BCAS4	-1.753649873	0.009163846
PRCP	-1.213020719	0.009163846
CYSLTR2	-1.962348901	0.009163846
RND2	-1.658033013	0.009163846
LIMD2	0.943719494	0.009244722
ERRFI1	-2.384789006	0.009244722
HFE	-1.185100616	0.009459457
ID3	-1.487077503	0.01009247
NKG7	-1.00503406	0.010474216
PID1	-3.518974859	0.011217588
UACA	-1.638832715	0.011217588
SELENOP	-1.615090202	0.011339222
AKAP12	-2.341153612	0.011673985
PLXDC1	-0.91746349	0.012102598
CBS	-2.339415872	0.012529786
FLNB	-2.058294158	0.01277455
PFN2	-1.683579338	0.013436255
OSGIN1	-1.033263711	0.013436255

ANG	-1.653475515	0.013436255
BOLA-DRA	-2.121241525	0.013436255
PYCR1	-2.106475896	0.013444373
LRRC71	-2.126244736	0.013444373
SLAMF7	-1.503465165	0.013508248
SCPEP1	-0.80876246	0.013904092
FZD1	-2.574484165	0.013904092
ASRGL1	-0.836681081	0.013904092
HMOX1	-0.923759378	0.014037639
SELENOM	-2.670218812	0.014037639
BMP1	0.905275382	0.014037639
C3AR1	-1.888394488	0.014954226
PLAC8B	-1.065040946	0.015528379
APBA1	-1.022479041	0.01630265
RCN3	-1.960956935	0.01630265
COL5A1	-2.996894094	0.017035872
TMEM140	-1.153706266	0.017843896
PDE7B	-1.255979378	0.017890939
ADA2	-1.275693381	0.018164386
ENSBTAG00000053153	-0.829628291	0.019185946
XBP1	0.899223238	0.019464341
ADAM15	-0.933420144	0.019465747
C1QB	-1.648417901	0.019820681
MXD4	-1.010256561	0.020223796
ISG15	-1.887363855	0.020337728
NACAD	-2.548758082	0.020380914
CTNNAL1	-2.481376268	0.020523383
PPP1R3C	0.995429336	0.021698485
CLIP3	-1.516390584	0.021698485
PLEKHA5	-2.019447791	0.024032528
CDKN2A	-2.128818738	0.024669104
DZIP1	-2.819249683	0.024672457
MYL9	-1.325539509	0.024680824
ENSBTAG00000052578	-1.641676782	0.024712726
TBC1D12	-0.900612778	0.02501733
STARD13	-1.902468385	0.025176851
ADAP2	-0.96860327	0.025176851
SHF	-2.388702037	0.025176851
KLF4	-2.216384997	0.02517832
ANKRD1	-4.098592323	0.02517832
TH	-1.733021333	0.025242408
F2RL2	-0.877394854	0.025242408
BGN	-1.071776679	0.025820942
SERPINE2	-4.594518388	0.025820942
CREG1	-0.88574911	0.026484246
HEBP2	-2.111407828	0.027046829
ADGRE5	0.819006444	0.027468564
CYP4V2	-1.214721206	0.027468564
CTNS	-0.898636335	0.027468564
SPATA20	-1.242787248	0.027741016

DSEL	-1.89467676	0.028239988
FADS6	-0.783449741	0.028318966
FAM13A	-1.095898026	0.028762327
CCDC152	-1.689622993	0.029094243
TNS1	-1.469509349	0.029094243
NOP53	-0.803506447	0.029158979
SLC25A6	-0.804223565	0.029251162
EHD1	-2.277387752	0.030223215
PLCD1	-1.064970215	0.03033958
MXD1	0.909314627	0.030586883
PTEN	0.961307128	0.030586883
IRX3	-2.145182937	0.03066702
KLHL22	-1.188013065	0.03066702
FSTL3	-1.44349831	0.031224733
SAMD11	-2.316576669	0.03128133
TINAGL1	-3.251705671	0.031722044
FOXO4	-1.114488649	0.031907583
ASAP3	-1.310512247	0.031957246
MARCKS	0.850194304	0.032763396
DENND1C	-0.813378048	0.033320309
BATF3	0.995341054	0.034354153
GLIS2	-2.043446104	0.035867597
C15H11orf96	-1.642826556	0.036236678
DEPDC7	-0.836227611	0.038006575
PCOLCE	-1.663974616	0.038704761
PECR	-1.599207896	0.03917755
LYN	0.853127488	0.040318338
ZFYVE21	-0.856132991	0.040318338
DST	-0.882971955	0.041213009
PLOD2	-0.942046148	0.041243642
CHAD	-1.371606237	0.041366021
CIITA	-2.556942165	0.041387138
NAAA	-1.158162842	0.041396256
PROS1	-1.975258552	0.041834419
FGF1	-2.452405629	0.042848504
FCGR3A	-1.341288346	0.043691843
LAPTM4B	-0.883136729	0.043944037
ANG2	-1.514907238	0.044305255
BCL9	-1.404636474	0.045362359
PTMS	-0.739699922	0.045867894
UTP14A	-0.746523109	0.045867894
SLC29A3	-0.744616061	0.045867894
SELENBP1	-1.156474815	0.047953027
SYTL2	-3.060304586	0.047953027
DCPS	-0.95646893	0.048367729
LMO2	-0.981373019	0.048971947
BLA-DQB	-1.287168178	0.049716564
TGFBR2	-0.710912414	0.049716564
FLT1	2.886393074	9.34E-12
F3	2.253714581	6.83E-10

IL1B	2.497077948	1.54E-09
NLRP12	3.105934541	1.64E-09
SLC28A3	4.509528908	4.71E-09
MT1A	4.19744298	2.33E-08
IL1R2	4.433029409	4.81E-07
TNIP3	1.808854437	4.81E-07
CXCL2	2.857026553	4.81E-07
VLDLR	1.625030271	4.81E-07
SLAMF1	3.028522499	4.81E-07
IL6	2.439123315	5.77E-07
IL1A	2.386517179	5.78E-07
SOD2	2.006692988	1.20E-06
MT2A	2.129781283	1.60E-06
TCN1	3.038189185	1.67E-06
CSF3	3.391831738	1.69E-06
SCNN1D	1.959582056	1.71E-06
MMP1	3.953000891	1.78E-06
ENSBTAG00000048500	1.780861099	2.50E-06
M-SAA3.2	1.765146659	6.00E-06
MMP3	4.124665146	7.00E-06
QPCT	1.721595772	7.03E-06
SLC39A8	1.901562879	1.45E-05
LOC281376	2.516197362	2.45E-05
CXCL3	1.373162116	2.55E-05
RUBCNL	1.616755088	2.68E-05
IL2RB	3.247891406	3.27E-05
LVRN	2.60626441	5.16E-05
AVIL	3.110193946	5.46E-05
CCL3	2.09799808	7.19E-05
PVR	1.558226766	9.75E-05
CCL4	2.399132912	0.00012623
RASD2	1.809427961	0.000155772
ABCG1	2.504101413	0.000176344
ENSBTAG00000053991	2.825304416	0.00021599
SLC44A3	2.084636571	0.000216722
GRO1	1.164518666	0.000223155
EREG	1.736670297	0.000427424
IL17REL	2.413598411	0.000521489
CCBE1	1.663454158	0.00056838
RETN	1.851602151	0.000732294
SAA3	1.697632224	0.0007385
MEFV	1.470195012	0.000794141
LPAR3	1.671451862	0.000879096
OCSTAMP	1.568910561	0.00089103
STC1	3.382270443	0.000998122
EHF	1.922918121	0.001048099
IL33	1.845822053	0.001099749
FAM8A1	1.764283041	0.001200239
GLT1D1	2.168758718	0.001219452
MMP12	1.376680654	0.001477321

DTX4	1.18051186	0.001590385
CXCL8	1.60180183	0.002025572
SLC16A6	1.418556026	0.002152416
EDN1	2.999713731	0.002153205
CCDC50	1.157322966	0.002377109
LOC112441463	3.092322546	0.002483582
CHI3L2	2.513736799	0.002503925
CCL8	2.513403862	0.002928834
UPP1	1.264621023	0.003186347
ITGB3	1.041018785	0.003581659
CLDN1	2.299005774	0.003970935
SERPINB2	1.414319575	0.0040066
SAMSN1	1.214186915	0.004427759
WFDC18	4.680218139	0.005199935
SAA2	1.576529683	0.005319944
ENSBTAG00000002290	1.627922259	0.005595403
S100A8	1.329340786	0.005789248
MT1E	2.263410428	0.006160446
CCL2	1.604323033	0.006196897
GJB2	2.390214505	0.006551039
AREG	1.739129814	0.007438232
TRPC6	1.831282376	0.007478696
TGM3	1.705641728	0.007666317
CYP3A4	1.411155583	0.007861517
DCSTAMP	2.074269734	0.00860349
HBEGF	1.249361477	0.008843556
ENSBTAG00000046944	2.413535548	0.009163846
CCL24	1.802079784	0.01009247
LAT	1.423664518	0.012969186
CYP27B1	1.814463903	0.013904092
CXCL5	1.25874936	0.016098765
CYP1B1	1.81975249	0.016437174
CRYBG1	1.067941794	0.017229412
ENSBTAG00000052099	2.392423536	0.019465747
KCNJ15	2.090152141	0.019820213
SLC2A3	1.102961051	0.020223796
HCST	1.536476997	0.020523383
LOC100848100	2.39779933	0.020523383
MST1R	1.528945072	0.021698485
C3H1orf162	1.009064692	0.02446644
PTGS2	1.864328188	0.024654907
CISH	1.535777984	0.025176851
SPRY4	1.415195228	0.027741016
GATA3	2.233441922	0.029094243
BACE2	1.32839339	0.029542115
ENSBTAG00000048591	2.019999818	0.03029215
CCL5	1.163985965	0.030586883
CA12	1.257005836	0.031810548
WNT5A	1.780339917	0.033932347
CSPG4B	1.305228555	0.034465188

TMEM170B	1.258799296	0.036848842
S100A9	1.070059666	0.03717073
ADGRF1	1.448328972	0.038704761
MT1E	1.580971812	0.038704761
A4GALT	1.559984085	0.043671509
RAPH1	1.144449907	0.044925612
SLC16A7	1.274368133	0.046255985
TES	1.07361187	0.047144905
DEFB7	1.328441011	0.048349947
RGS16	1.043315182	0.048367729
