# Supplementary material for: Lipopolysaccharide triggers different transcriptional signatures in taurine and indicine cattle macrophages: Reactive oxygen species and potential outcomes to the development of immune response to infections
Source: PLoS One. 2020 Nov 6;15(11):e0241861. doi: 10.1371/journal.pone.0241861 (PMC7647108; doi:10.1371/journal.pone.0241861)
Supplement: S4 Table — Differential expression was performed on RNA sequencing data from unstimulated MDMs between Holstein and Gir breeds. genes that showed statistical differences in contrast (LogFC≥1; CPM>1; FDR<0.05) are shown. (PDF) [file pone.0241861.s006.pdf]

Gene Symbol	logFC	FDR
IFITM1	-7.852275065	3.11E-21
SYT4	-5.26669844	1.18E-16
PTGR1	-3.135921914	4.09E-11
LOC100335553	-8.230478674	7.01E-11
TREML2	-3.260636022	2.38E-09
CD38	-4.781830701	4.65E-09
ENSBTAG00000052571	-5.699317133	6.37E-08
ABCB1	-3.877014609	9.59E-08
BOLA-DQA2	-6.876830923	2.44E-07
OAS1Z	-4.120949185	3.19E-07
ENSBTAG00000052720	-2.262659445	1.07E-06
SLC28A3	-4.068383361	5.40E-06
MMP25	-2.892210532	6.65E-06
ENSBTAG00000051047	-2.702560476	1.35E-05
PTGDS	-7.292770384	2.19E-05
BOLA-DQA5	-6.164085263	2.53E-05
CLU	-2.514248108	3.12E-05
NDUFA4L2	-2.472763934	0.000157067
TSPAN13	-2.502503447	0.000196427
SAA2	-6.253447126	0.000335877
EGLN3	-1.976325375	0.000645484
SDS	-1.95521561	0.001422591
BRB	-3.507406063	0.002503665
LPL	-5.564090846	0.004036144
TNK1	-2.530141379	0.005117445
IL1RN	-2.508213426	0.008895861
PLD4	-2.719002108	0.009566418
CDH23	-2.833109128	0.012663944
RRAGA	-1.201713107	0.012663944
COMMD6	-1.862954903	0.012872715
FABP3	-2.313924024	0.013903456
OCSTAMP	-2.614863013	0.014133593
JAML	-1.960861247	0.014329712
SEC23A	-1.669863374	0.014987564
LOC100139670	-2.266168778	0.015204587
RSAD2	-3.18225624	0.015255953
SLC13A5	-1.562267114	0.016404993
TMEM150A	-1.568342611	0.017715912
CDH5	-2.797046215	0.018038996
ECE1	-1.456335374	0.018038996
CA4	-4.819364637	0.019492217
MGLL	-2.734960821	0.020627217
ENSBTAG00000052369	-2.234418053	0.020627217
BMPR1A	-2.051402771	0.021741863
SLC13A3	-2.448913672	0.021741863
ENSBTAG00000052578	-3.071451558	0.024602173
ACSL4	-2.13819498	0.025866969
PTGS2	-2.562265973	0.02628396
TUB	-2.283572189	0.026349091

CSPG4B	-1.535313566	0.031648873
SCIN	-4.58699819	0.040692148
CCR5	-1.904155556	0.046763331
DNAJC6	-1.927178103	0.046841337
CTSK	-1.434988472	0.047143148
BOLA-DQB	-3.493131468	0.047143148
SLC7A11	-1.876550522	0.047143148
MICU3	-1.658795377	0.048952433
CD180	-2.350435423	0.048952433
BOLA-NC1	-1.469429447	0.049330159
ATP8B1	-1.59618813	0.049330159
JAM2	-2.162646692	0.049330159
TGFBR3	-3.64871078	0.049330159
AOX1	8.222296298	7.80E-36
ENSBTAG00000020684	4.471207149	3.30E-21
LOC539009	6.027647915	1.74E-18
ENSBTAG00000048514	6.559988194	8.79E-13
ENSBTAG00000053827	4.638415225	2.24E-12
RFLNA	5.02971867	4.65E-09
ENSBTAG00000034871	3.253596532	1.85E-08
LOC526163	4.73953985	2.91E-08
ENSBTAG00000027412	2.982091764	8.66E-08
ENSBTAG00000049212	5.675285603	1.64E-07
MARCO	3.635458723	2.63E-07
CREB3L3	6.516651556	2.69E-07
LOC515676	4.044516488	5.49E-07
FCER2	3.470202086	8.51E-07
PTER	2.064650477	8.63E-07
ENSBTAG00000040564	4.54428365	1.61E-06
ENSBTAG00000038893	4.226825403	2.60E-06
TAGAP	2.45277623	3.32E-06
LOC529196	2.283573364	3.89E-06
ENSBTAG00000049207	4.554760782	4.36E-06
LOC504858	2.630820885	1.35E-05
CRYM	4.026952868	5.23E-05
SLC35A1	1.664068752	5.99E-05
ENSBTAG00000039413	2.356207057	6.75E-05
SDR42E2	3.75556275	0.000147008
ENSBTAG00000032217	2.318893117	0.000198421
TRPC6	3.163566165	0.000198421
ENSBTAG00000053097	1.87185832	0.000213312
HACD4	2.203098565	0.000246748
ENSBTAG00000040367	3.682953825	0.000256896
ENSBTAG00000048616	2.362895685	0.000419
ENSBTAG00000027075	2.015117542	0.000756152
FCAR	1.538727125	0.001089748
CAPN3	1.974232184	0.001089748
ENSBTAG00000007296	3.018887976	0.001093811
LOC107131287	3.946909097	0.001183874
ASF1B	2.132890413	0.001450227

DMKN	3.185950367	0.001465369
ENSBTAG00000045689	1.846201589	0.001618282
CLEC3B	2.28778829	0.001621725
CALY	2.973101867	0.001854361
ZNF385A	1.376794903	0.001947313
PCNA	1.682852946	0.002339663
GGCT	1.637366236	0.002460439
ENSBTAG00000050015	2.61037285	0.002503665
TCF19	2.495947189	0.00323432
UNC93A	2.586876677	0.003983291
CDCA5	2.246510005	0.004290332
CCHCR1	1.694423934	0.00432986
E2F1	1.8232299	0.004635947
LRRC1	2.667720522	0.004790262
ENSBTAG00000046383	1.948588765	0.005117445
ENSBTAG00000026909	2.56789522	0.005201402
CARD11	2.680750293	0.005216285
ENSBTAG00000034662	2.663458946	0.005396663
LOC101904667	1.468562364	0.005853425
TGM3	3.160457674	0.006656079
ENSBTAG00000047029	1.770644152	0.011167321
CDC25B	1.435683184	0.011533531
HAVCR2	1.918034739	0.012663944
POLE	2.140823898	0.013131044
PHF19	1.345391025	0.014329712
PADI4	1.958870454	0.014329712
SAPCD2	2.199200612	0.014329712
HIRIP3	1.360116815	0.016404993
ENSBTAG00000037925	2.547909611	0.01658066
LIG1	1.540237887	0.016702409
ENSBTAG00000025283	2.37202871	0.016878424
SMC2	1.732639526	0.018038996
ENSBTAG00000022275	1.708697782	0.019492217
KIF14	1.896708941	0.019519012
CLSPN	1.926702249	0.020627217
LOC100196897	2.755416064	0.020627217
SUV39H1	1.206776118	0.020627217
LOC101905222	2.619961944	0.021741863
P2RY11	2.103744048	0.021741863
EZH2	1.247692455	0.022289806
ELN	1.744147048	0.022490867
RAD51AP1	1.912540996	0.022490867
RBL1	1.610180745	0.025260947
CCNF	1.472607421	0.025848856
RECQL4	1.846794674	0.026349091
INCENP	1.322497792	0.028512082
PLK4	1.475291707	0.028596064
HSF2BP	2.056399218	0.03075345
THEM6	1.975127639	0.030795658
E2F8	2.674550857	0.030944504

ARHGEF39	1.653193523	0.031126949
BFSP2	1.955179242	0.031648873
NMRAL1	1.223594313	0.033690318
RARRES1	1.465168434	0.034508334
NSD2	1.380779947	0.036830639
LOC404051	2.464472717	0.04013683
TUBB2B	1.390983733	0.040692148
POLD1	1.385859632	0.042800648
HAPLN4	2.860535872	0.043213052
CDKN3	2.25317927	0.043213052
KIF15	2.127601377	0.044371824
LMNB1	1.699109411	0.046763331
GPR19	2.205532724	0.047143148
CPT2	1.063879687	0.049330159
