# Supplementary material for: Lipopolysaccharide triggers different transcriptional signatures in taurine and indicine cattle macrophages: Reactive oxygen species and potential outcomes to the development of immune response to infections
Source: PLoS One. 2020 Nov 6;15(11):e0241861. doi: 10.1371/journal.pone.0241861 (PMC7647108; doi:10.1371/journal.pone.0241861)
Supplement: S5 Table — Differential expression was performed on RNA sequencing data from LPS treated (100 ng/ml) MDMs between Holstein and Gir breeds. Genes that showed statistical differences in contrast (LogFC≥1; CPM>1; FDR<0.05) are shown. (PDF) [file pone.0241861.s007.pdf]

Gene Symbol	logFC	FDR
SYT4	-6.527153	2.64E-31
PTN	-7.874586	3.92E-30
IFITM1	-8.774852	4.33E-29
CA4	-9.946424	1.28E-23
DCN	-9.399865	1.84E-23
HTRA3	-6.87344	6.35E-21
PDGFRA	-7.059054	1.94E-20
PTGR1	-3.910481	2.07E-20
ABCB1	-5.458288	3.69E-20
LOXL1	-5.257463	8.37E-20
TCAM1	-5.844489	2.45E-19
FMOD	-7.527484	2.90E-19
HHIPL1	-6.538891	2.26E-18
S100A16	-6.543599	2.99E-18
PXDN	-7.692579	6.66E-18
DPYSL3	-6.373627	6.66E-18
PRELP	-8.246706	7.70E-18
SERPINH1	-5.310758	2.72E-17
KIAA1217	-6.817127	1.63E-16
PABPC5	-7.10083	2.31E-16
MMP25	-3.037792	3.21E-16
S100A14	-6.589963	3.21E-16
FSTL1	-5.030471	3.95E-16
PTPN13	-6.404404	8.54E-16
EPAS1	-5.598882	1.13E-15
SNAI2	-7.021426	1.27E-15
LAMA4	-5.942843	4.13E-15
ENSBTAG00000051047	-3.646488	4.36E-15
RAMP3	-5.056007	5.82E-15
PRRX2	-6.061922	8.37E-15
TSPAN13	-3.260211	1.47E-14
LOX	-6.182639	1.53E-14
CREB3L1	-6.119371	1.57E-14
HAPLN3	-5.249015	2.40E-14
MAP1A	-4.606091	2.54E-14
NT5E	-6.325458	2.91E-14
COL3A1	-7.901375	6.75E-14
WWC1	-6.424333	8.14E-14
KCTD15	-5.869279	1.35E-13
FBLN2	-6.504414	1.60E-13
PLAT	-5.41787	2.22E-13
NID1	-5.672903	2.39E-13
NDUFA4L2	-3.28071	2.71E-13
GJA1	-5.570591	2.86E-13
AXL	-3.965685	4.51E-13
CCN1	-5.539708	4.67E-13
LOC100335553	-5.825172	5.37E-13
MYH10	-3.509976	1.17E-12
RAB34	-4.792741	1.28E-12

ENSBTAG00000045507	-4.831466	1.35E-12
LOXL2	-7.080388	1.35E-12
LOC100850808	-4.79232	1.59E-12
FGFR1	-5.258592	1.85E-12
SLFN11	-6.051609	2.88E-12
C1QTNF1	-5.654116	3.20E-12
NXPE4	-6.601618	3.25E-12
HS3ST2	-6.50478	3.64E-12
SRPX	-5.899227	6.24E-12
DENND2B	-4.288084	6.73E-12
FBLN5	-5.917813	6.78E-12
LAMB2	-4.249651	7.68E-12
PCOLCE2	-6.848012	7.70E-12
CAVIN1	-3.986298	7.71E-12
SERPINE1	-4.779419	9.56E-12
MEDAG	-5.612765	9.74E-12
GPC4	-5.900153	9.98E-12
EFEMP2	-4.326142	1.39E-11
C1S	-5.192145	1.72E-11
ENSBTAG00000052571	-4.931413	1.82E-11
PTPRF	-2.866939	2.28E-11
MAP1B	-3.315886	2.64E-11
CEMIP	-6.282785	2.89E-11
ARAF	-3.05438	2.90E-11
TBXA2R	-4.621002	3.21E-11
SULF2	-3.535386	3.31E-11
SCARF2	-4.887632	3.33E-11
HSPB8	-5.742456	3.85E-11
COL1A1	-6.536476	3.98E-11
IL1RL1	-6.025432	4.19E-11
CD38	-4.391328	5.19E-11
NTN4	-6.407881	5.44E-11
PRSS23	-4.196717	5.48E-11
ARHGAP29	-5.409053	7.25E-11
COL1A2	-6.528006	7.28E-11
EHD2	-4.976829	7.32E-11
CDH11	-4.603696	8.28E-11
FBLIM1	-4.803987	9.58E-11
TNC	-6.675481	1.00E-10
CD248	-6.079365	1.29E-10
ENSBTAG00000001051	-5.790635	1.53E-10
NFIX	-4.688944	1.58E-10
LGR4	-5.475055	2.23E-10
ERRFI1	-2.612256	2.41E-10
DKK3	-5.256833	2.76E-10
PTGDS	-9.930097	3.07E-10
CRISPLD2	-4.102862	3.47E-10
CDA	-4.316267	3.72E-10
SPARC	-5.843782	3.86E-10
FST	-5.278823	4.50E-10

TENM3	-5.72592	4.69E-10
MEIS1	-5.572239	6.86E-10
PTK7	-3.888926	1.20E-09
PDGFRB	-4.456389	1.40E-09
LUM	-6.23026	1.53E-09
MMP2	-6.465675	2.62E-09
FBN1	-6.001486	3.25E-09
AMOTL2	-3.254378	3.49E-09
TAGLN	-5.788433	3.65E-09
NDN	-4.583047	3.91E-09
SOCS1	-2.2732	5.55E-09
JAML	-4.048064	5.82E-09
MGLL	-3.255851	6.67E-09
GPR68	-4.168884	7.86E-09
PDLIM1	-3.933695	8.67E-09
GEM	-3.587558	9.77E-09
ENSBTAG00000052720	-2.128605	1.04E-08
PRRX1	-5.452668	1.07E-08
JUP	-3.418202	1.34E-08
ALDH1A3	-3.237326	1.38E-08
FRMD6	-3.564118	1.47E-08
SMO	-3.73227	1.66E-08
LIPG	-4.895911	1.89E-08
TIMP3	-4.027338	2.99E-08
PALLD	-3.295685	3.19E-08
CCN2	-5.38843	3.39E-08
CHI3L2	-2.572119	3.68E-08
PI3	-4.324291	3.73E-08
FLNC	-5.186235	4.50E-08
CDC42EP1	-2.070183	4.51E-08
LOC104968478	-2.536445	4.76E-08
COL6A2	-5.843027	4.77E-08
LTBP4	-3.11625	5.03E-08
VEGFA	-2.121576	5.83E-08
CDO1	-3.226673	5.97E-08
CSF1	-3.118988	6.03E-08
FHL2	-4.670551	6.05E-08
SGCE	-4.159861	7.05E-08
CAV1	-4.861757	8.67E-08
CFB	-2.970922	9.76E-08
TRIM2	-3.644665	1.56E-07
BOLA-DQA2	-6.597922	1.84E-07
TMEM119	-4.003654	1.89E-07
CDH2	-4.611061	2.20E-07
CLU	-2.562518	2.33E-07
PLEKHA5	-2.739597	2.57E-07
ENSBTAG00000001219	-1.71869	2.68E-07
CERCAM	-3.044222	3.18E-07
SAA2	-7.013202	3.30E-07
SFRP4	-4.18088	3.46E-07

IGFBP6	-2.396333	3.83E-07
PTPRS	-2.649872	5.19E-07
CCDC136	-2.785642	5.54E-07
COL6A1	-5.676119	5.98E-07
ENSBTAG00000053401	-3.577966	6.42E-07
TPM2	-3.218081	6.49E-07
ADORA2A	-1.753426	7.06E-07
CCR5	-2.646784	7.06E-07
OLFML3	-3.202358	7.40E-07
COL5A2	-6.305519	7.62E-07
LRRC32	-4.112439	8.20E-07
GBP5	-2.192079	9.83E-07
NCKAP1	-3.031738	1.01E-06
JAM2	-2.885602	1.15E-06
SERPINE2	-5.514103	1.18E-06
KLF4	-2.313757	1.25E-06
TNK1	-2.938285	1.30E-06
SLC39A14	-2.934823	1.49E-06
CDH15	-2.862665	1.55E-06
SAA3	-1.781343	1.59E-06
DZIP1	-2.692882	1.73E-06
FKBP10	-2.653343	1.80E-06
LOC511531	-2.155326	2.16E-06
CDH23	-2.47431	2.52E-06
SLC28A3	-2.917442	3.09E-06
TREML2	-2.681392	3.76E-06
ASNS	-2.46147	3.97E-06
ALDH1L2	-2.951239	4.49E-06
AVIL	-3.571521	4.86E-06
MXRA8	-3.430226	5.07E-06
MEGF6	-3.127859	5.07E-06
OAS1Z	-3.850876	5.97E-06
HOXB3	-3.230134	6.23E-06
NUAK2	-2.733108	6.49E-06
NCF1	-1.452044	6.94E-06
FSCN1	-2.330195	7.72E-06
EHD1	-2.314021	8.60E-06
FERMT1	-2.651263	8.76E-06
RAB13	-2.426733	9.24E-06
TGM1	-2.216378	9.62E-06
BATF2	-1.798295	1.03E-05
TMEM158	-1.826575	1.11E-05
FN1	-3.507389	1.21E-05
KANK2	-2.179905	1.26E-05
IL36A	-1.512113	1.39E-05
CSPG4	-2.806034	1.72E-05
SLC13A5	-1.853986	1.81E-05
PID1	-3.886624	1.88E-05
TUB	-2.61617	1.88E-05
FLNB	-2.232946	1.89E-05

GSTM2	-2.754957	1.91E-05
LOC100139670	-3.037657	2.15E-05
LOC112443175	-1.577894	2.26E-05
SELENOM	-2.845859	2.56E-05
PTPRM	-3.120519	2.66E-05
MFGE8	-2.021567	2.69E-05
BCL2A1	-1.498207	2.69E-05
TNFRSF12A	-2.00207	2.74E-05
AFAP1	-2.195029	2.81E-05
ENSBTAG00000000109	-2.398513	3.04E-05
BOLA-NC1	-1.657665	3.46E-05
NEO1	-1.739936	4.53E-05
PIM3	-1.527909	4.93E-05
ENSBTAG00000009111	-2.297125	5.33E-05
IRF1	-1.275874	5.54E-05
CCRL2	-1.63553	5.81E-05
RUNX2	-2.200939	5.99E-05
MAP2K6	-2.965176	6.33E-05
PADI6	-2.394771	6.48E-05
STXBP1	-1.963064	6.81E-05
MPZL3	-2.210705	6.99E-05
ECE1	-1.761489	7.04E-05
VSIR	-1.322117	7.06E-05
ESM1	-3.98662	7.99E-05
NID2	-4.378724	8.34E-05
ZNF8	-1.484943	8.50E-05
COL5A1	-2.913977	8.57E-05
CCDC85C	-1.694906	8.90E-05
RNF135	-1.242551	9.01E-05
RRAGA	-1.183944	9.13E-05
CTNNAL1	-2.259693	9.40E-05
CSRP2	-3.528078	9.92E-05
CD82	-1.812106	0.0001003
YES1	-3.272912	0.0001109
NUMBL	-2.186745	0.0001112
MARK1	-2.358851	0.0001367
COL12A1	-5.369824	0.0001396
TFPI2	-2.267932	0.0001468
BEST1	-1.821924	0.0001684
CXCL3	-1.158397	0.0001862
VSTM4	-2.601598	0.0001955
PLXDC1	-1.951781	0.0001958
LOC507055	-2.124944	0.0002056
LIPM	-2.962923	0.0002123
MYOF	-2.098674	0.0002259
SHF	-2.253648	0.0002625
DDAH2	-2.364636	0.0002701
LOC526769	-2.139453	0.0002758
FAT1	-3.215764	0.0002767
CHST2	-2.401076	0.0002767

ADSS1	-1.788569	0.0002902
GGT5	-1.581536	0.0003049
TLN2	-1.856846	0.0003226
FAS	-2.101273	0.0003263
RPP38	-1.874995	0.0003393
IL27RA	-1.694712	0.0003575
ADARB1	-2.0981	0.0003806
ASCL2	-2.331493	0.0003813
ID3	-1.702451	0.0003843
QSOX1	-2.023402	0.0003843
GATA3	-1.819225	0.0003855
MCOLN2	-1.909317	0.0003888
ENSBTAG00000054678	-1.554469	0.0003935
ENSBTAG00000054514	-1.216773	0.0003935
TMEM150A	-1.909839	0.0003968
PDE5A	-2.129367	0.0004052
PTAFR	-1.112218	0.0004194
ENSBTAG00000051191	-1.19575	0.0004251
ENSBTAG00000001476	-2.040789	0.000445
POSTN	-3.867322	0.0004534
ENSBTAG00000053667	-2.505292	0.0004582
WARS1	-1.3889	0.0004778
APLP1	-1.62305	0.000501
NDRG2	-1.341002	0.000501
ZMYND15	-1.497755	0.0005294
TRMT44	-1.28755	0.0005371
TEF	-1.233224	0.00054
MYO1D	-1.450289	0.0005503
ITM2B	-1.196626	0.0005747
CHCHD10	-1.38182	0.0006086
NUAK1	-3.291974	0.0006432
MTHFS	-1.143072	0.0006432
ENSBTAG00000049968	-1.194151	0.0006513
BMF	-1.346834	0.000654
VNN1	-2.482093	0.0006669
JSP.1	-1.76114	0.0007018
HSPB6	-1.835776	0.0007167
CRIM1	-2.111607	0.0007225
PECR	-2.009337	0.0007701
NOTCH3	-2.530084	0.000772
RCN3	-1.583376	0.0007807
KLF2	-1.476154	0.0008142
NOS2	-2.830151	0.0008554
MT1E	-1.934994	0.0008595
TTC21B	-1.915932	0.0008693
CACNA1A	-2.12205	0.0008719
ASAP3	-1.811481	0.000927
ENSBTAG00000051307	-1.159664	0.0009468
LOC512486	-1.24716	0.0009499
VASN	-1.335765	0.0009676

GPR183	-1.689983	0.000968
LOC507581	-1.258155	0.0009905
KIF7	-2.163496	0.0009992
TAP1	-1.274536	0.0010368
H2BC5	-2.065241	0.0010475
DGKD	-0.982948	0.0010664
BCL2L11	-1.40473	0.0011214
DNAJA4	-1.271761	0.0011856
LOC100298356	-1.717928	0.0013124
OLR1	-2.980395	0.0013165
MIR6518	-1.369405	0.0013303
NEDD4L	-1.631379	0.0013487
ANO9	-1.993567	0.0013548
TIAM2	-1.617339	0.0013731
TINAGL1	-3.198591	0.0013781
TMCO4	-1.151238	0.001386
PYCR3	-1.110463	0.0014071
DHRS1	-1.146423	0.0014126
PRODH	-1.873255	0.001415
LONP2	-1.091144	0.001415
COMMD6	-1.584829	0.0014328
GTF3C1	-1.03955	0.0014774
PECAM1	-2.067537	0.0014832
ENSBTAG00000002088	-1.26653	0.0015081
MTMR7	-1.695517	0.0016224
IPCEF1	-1.757045	0.001812
ADM	-1.372148	0.0018497
TMEM140	-1.488321	0.00193
ENSBTAG00000046633	-1.243403	0.00193
SLC25A45	-1.678166	0.0019466
TMEM215	-2.494442	0.0019761
DNAJC6	-1.780827	0.0020288
FAM13A	-1.262439	0.002112
RSAD2	-3.397639	0.0023401
IL1RN	-3.155851	0.0023505
GLIS2	-1.534569	0.0024338
ITGA9	-3.187471	0.0024988
CBS	-1.959265	0.0025298
LOC520336	-2.4883	0.0025731
PLBD1	-1.983398	0.0026486
SLC25A23	-1.153967	0.0026486
TM4SF19	-2.176452	0.0026533
FEZ1	-2.296286	0.0026986
MAPK11	-1.635085	0.0027029
LOC784541	-1.50531	0.0027901
EGLN3	-1.543488	0.0028537
PTMA	0.9341925	0.0028722
SYN1	-1.342592	0.0028816
CDCP1	-2.031095	0.0031439
BHLHE40	-1.107131	0.003148

HIVEP1	-1.37456	0.003148
TCF7L1	-2.089415	0.0031684
CREG1	-1.322342	0.0032034
SLC25A19	-1.03193	0.003258
MYL9	-1.37868	0.0033131
ENSBTAG00000051168	-1.95886	0.0033676
LOC616942	-1.265283	0.0033725
HIF1A	-1.335913	0.0034323
TLE5	-1.154549	0.003463
GDNF	-2.219797	0.0034874
ENSBTAG00000048049	-1.353225	0.0035553
C11H9orf50	-1.43495	0.0036294
KDM8	-1.302767	0.0039072
IGSF6	-0.980854	0.0040604
AGMAT	-1.645525	0.004082
PDHA1	-1.003183	0.0041012
ANKH	-1.434461	0.0042489
RFC2	0.9402676	0.004249
GNB3	-2.056979	0.0043908
SDC1	-2.266295	0.0044645
TMEM97	0.9420181	0.0045505
NAPRT	-1.2365	0.0046241
NINJ1	-1.067676	0.0046241
OPLAH	-1.235159	0.0046376
NR1D1	-1.145242	0.0046489
MVB12A	-0.983154	0.0046489
CBLN3	-1.417175	0.0047014
TSC22D3	-1.026265	0.0047632
EBI3	-1.359381	0.0049791
PROS1	-2.124858	0.005125
ARRDC4	-1.197895	0.0052108
SLC13A3	-1.807795	0.0052108
COL6A3	-1.130826	0.0053451
ANKRD22	-2.150235	0.0056419
RNF157	0.8944879	0.0056485
TSHZ3	-2.018127	0.0057178
ANKRD2	-1.67639	0.0058579
CD81	-1.009114	0.0059371
DSEL	-1.612894	0.0059371
ADGRA3	-2.106684	0.0059747
CFL2	-1.475112	0.0063091
OCSTAMP	-1.5333	0.0064002
ENSBTAG00000048982	-1.644914	0.0065242
PRRG2	-2.180564	0.0066798
GBP4	-3.224176	0.0066798
CTH	-1.878562	0.0069374
EHF	-1.11609	0.00703
SASH3	0.9788404	0.0070337
CLMN	-1.754846	0.0071236
CLEC4E	-1.565578	0.0071721

HDHD3	-1.4812	0.0072565
NEURL1	-1.163383	0.0076426
CASKIN2	-1.187301	0.0076799
HSF4	-1.327004	0.007722
LOC112441507	-1.827062	0.007767
C29H11orf24	-1.180244	0.0077746
CLBA1	-1.262105	0.0077754
CCND3	0.9203578	0.0078887
SLC46A1	0.9155222	0.0079644
FCAR	0.9400015	0.007968
TNFAIP3	-1.010858	0.0079995
PCNT	0.8935431	0.008038
CNTNAP1	-1.763651	0.0080435
GRK5	-1.301732	0.0081652
ARL13B	-1.483742	0.0081652
MCOLN3	-1.979454	0.0082374
ITGA1	-2.274627	0.0085511
KCNK6	-1.069195	0.0086172
CDC42EP4	-1.016412	0.0086793
MARCHF3	-1.425838	0.0087114
HMGA1	0.9227961	0.0087456
RHOH	-1.202129	0.0088525
PSD	-1.3729	0.0090396
CBLB	-1.892808	0.0091836
ATP6V0A1	-1.067766	0.0091836
PLBD2	-0.857633	0.0092904
ZDHHC7	-0.839411	0.0095628
BMPR1A	-1.72752	0.009657
PFKFB4	-0.834545	0.0097798
ADGRA2	-1.264583	0.0099808
H2AZ2	0.9097162	0.0100321
RPLP0	0.9552415	0.0102764
TOM1L1	-1.558532	0.0102814
NFE2L3	-1.701294	0.0103147
BCL9L	-0.858507	0.0103534
LOC508153	-1.005603	0.0104033
THEMIS2	-0.846357	0.0106316
ENSBTAG00000010722	-1.49331	0.0106368
TNS2	-1.036842	0.0106479
NFU1	-1.009508	0.010865
SYTL2	-2.18284	0.0113051
ERMARD	-1.103908	0.0113292
PLEKHA6	-1.664315	0.0113299
CORO1A	0.8608703	0.0113841
ABRACL	0.9891742	0.0116213
LOC535280	-1.297323	0.0117302
CASP2	0.825454	0.0119538
ENSBTAG00000049557	-1.298683	0.0125307
PANX2	-1.393097	0.0126238
ENSBTAG00000038797	-1.563152	0.0127404

DLG4	-1.700301	0.0132421
PER2	-1.082044	0.0132808
ATF3	-0.931035	0.0132808
MAPK12	-1.073483	0.0133939
SRL	-0.954284	0.01365
IFIH1	-1.242596	0.013838
ICAM1	-0.883554	0.013838
MARCKSL1	-0.993623	0.0141516
DBP	-1.579302	0.0141516
DDT	0.8633196	0.0141516
SLC25A33	-1.100134	0.0142229
ENSBTAG00000048772	-1.207941	0.0142939
EFHB	-1.638131	0.0143093
ARHGAP35	-0.918106	0.0145581
CCDC69	-1.297294	0.0145888
GJB2	-1.698026	0.0152403
HMGB1	0.8092342	0.015507
TUFT1	-1.334904	0.015781
PDE7B	-1.652488	0.0158731
LOC786372	-2.206278	0.0159598
ADAMTS10	-1.380551	0.0159705
MYBPH	-1.493447	0.0159705
TSPAN32	-2.614855	0.0159705
TYMS	0.9603942	0.016111
SULT1A1	-1.156656	0.0161121
C11H9orf16	0.9000704	0.0161909
ENSBTAG00000003598	-0.985519	0.0163069
GPT2	-1.251064	0.0163069
ABHD8	-0.862043	0.0163069
ROR2	-1.56295	0.0163384
ALDH16A1	0.8026474	0.0166556
NPR2	-1.13885	0.0166595
DSTN	-0.988185	0.0167928
SLBP	0.8050179	0.0168491
CYP4V2	-1.330342	0.0169057
LOC616254	-1.072466	0.0169531
CA11	-1.603876	0.0170375
BCAS4	-1.351385	0.0173458
CXCL16	-0.91849	0.0174575
TTF2	0.8447105	0.0174797
ENSBTAG00000003760	-0.979939	0.0176487
ENSBTAG00000051225	-1.413051	0.0178065
ZCCHC17	-0.804549	0.0179268
MYCL	-1.160365	0.0180011
GNAL	-1.283619	0.0181428
FLVCR2	0.8270042	0.0182705
KLHL8	-1.159019	0.0182742
ANTXR1	-1.70008	0.0183417
FERMT2	-1.473771	0.0184204
CDK5RAP2	0.9336074	0.0185542

DEK	0.8423906	0.0187697
IRAK2	-0.97664	0.0187712
NQO1	-0.930239	0.0187712
PHGDH	-1.257484	0.0189207
DOCK6	-0.869151	0.0190622
ABHD6	-1.453107	0.0190622
SLC11A1	-0.826352	0.0191441
ANKLE1	0.9163684	0.0192492
LRRC8E	-1.16569	0.0193948
CATSPERD	-1.110936	0.019517
MICU1	-0.847416	0.0197944
IL17RC	-0.841933	0.0199588
NLRX1	0.8028402	0.0202792
NFKBIE	-0.801376	0.0204869
NEK3	-1.081166	0.0205071
CEACAM19	-1.271738	0.0206235
VRK1	0.8501797	0.0209373
CSPG4B	-1.15017	0.0211338
IGHMBP2	-0.845158	0.0211654
NDUFA4	-1.070279	0.0211654
BCAR1	-0.857507	0.0211798
DTX3	-1.438449	0.0212033
NOD2	-0.997409	0.0212137
EPS15L1	-0.787258	0.0217243
RASGRP1	-1.577181	0.0217337
TNIP1	-0.947015	0.0217831
TDP1	0.9433594	0.0221585
GRAMD2B	-1.388097	0.022216
KIAA0513	-1.078543	0.0222987
PROKR2	-1.303612	0.0222987
KDM6B	-0.744409	0.0222987
DOCK2	0.8493434	0.0225785
HOMER3	-0.969414	0.0227511
NUP62	0.7720272	0.0227511
SLC3A2	-0.836387	0.0229054
PTGS2	-2.023581	0.0229212
SDC3	-1.321279	0.0233948
MADD	-0.804325	0.0234337
IMPDH1	-0.809434	0.0236137
NRROS	-1.046867	0.0236175
IGSF3	-1.634136	0.0238823
UPK3B	-1.294709	0.0246807
TEAD3	-1.007875	0.0247467
CA9	-1.737037	0.0247467
GPRC5A	-2.964785	0.0249979
MET	-1.888912	0.0251721
ZACN	-1.102992	0.0253911
SNAI1	-1.048854	0.0254312
TLR4	-1.089869	0.0258168
PARP3	-0.938967	0.0260961

SH3TC1	-0.817002	0.0260961
EHD3	-1.396382	0.0263048
NFKB2	-1.049477	0.0265483
ENSBTAG00000033107	-1.119756	0.0266317
LTBP3	-0.824597	0.026843
PAXIP1	0.8632337	0.026843
ADSL	0.7895548	0.026843
BBS4	-0.79993	0.026843
CDHR5	-1.079995	0.026843
ENSBTAG00000052099	-1.683186	0.026843
SIRT5	-1.156867	0.027104
CCDC80	-1.38394	0.0278027
ALDH2	-0.881561	0.0278727
BHLHE41	-1.408199	0.0278892
ENSBTAG00000051239	-1.051802	0.0281683
POLD3	0.8597826	0.0281683
PYCR1	-1.55138	0.0281775
TMEM63A	-0.859572	0.0282557
PDIA5	0.8847743	0.0283587
NCKAP5L	0.7627199	0.0284245
BUB3	0.7869207	0.0285075
ZNF503	-1.398675	0.0287368
MCPH1	0.8693023	0.028853
NXT1	0.9118549	0.0290299
SMOX	-1.047013	0.0292412
NCAPH2	0.8255677	0.0306169
TRIM65	-0.847179	0.0307637
MEIS2	-1.497727	0.0310414
TPPP	-1.287595	0.0311929
SDC4	-0.856129	0.0312931
TMEM106A	-0.867991	0.0314542
SIRPB1	-1.198959	0.0314542
RBBP8	0.8007803	0.0316522
NOL4L	-0.822813	0.0318909
CABP1	-1.607499	0.0318909
TPCN2	-0.758627	0.0319063
S1PR3	-1.978112	0.0321159
C11H9orf116	-0.978099	0.0322091
RANGAP1	0.8452047	0.0322751
RTN4	0.7174231	0.0323144
TCHH	-2.070531	0.0323315
ENSBTAG00000053696	-0.972993	0.0323315
FZD1	-1.312426	0.0323325
TMEM9	-0.86996	0.0323532
CCPG1	-0.994548	0.0323532
ELMO3	-2.302735	0.0325342
ENSBTAG00000039334	0.9270224	0.0325979
ENSBTAG00000051722	-1.668989	0.0325979
FAH	-0.88926	0.0328259
RGS20	0.9272554	0.0330843

ENSBTAG00000045948	-0.729631	0.0334539
SLC39A6	0.9412193	0.0337079
OGT	-0.767141	0.0340913
BOLA-DRA	-2.193819	0.034128
IL2RG	-0.815937	0.036217
NES	-2.138854	0.0366666
C7H19orf71	-0.951011	0.0366888
SLC2A10	-1.927563	0.0367087
PRDX6	0.7113897	0.0367087
RTN2	-1.296327	0.0367976
NACAD	-1.451031	0.0368346
IFT43	-1.447894	0.0369122
TNFAIP2	-0.878388	0.0370472
NXN	-1.020636	0.0371196
PSAT1	-1.075845	0.0372857
CEP78	0.8670507	0.0374188
BUD23	0.7432523	0.0374188
TM6SF2	0.8747579	0.0376343
SLAMF9	-2.423142	0.0379997
LTBP1	-1.93179	0.0379997
SDS	-1.03615	0.0383105
GABARAPL1	-0.963428	0.0385532
SORT1	-1.195452	0.0386094
LOC784451	0.9670425	0.0387226
MUTYH	0.9961018	0.038791
PLP2	0.7278129	0.0389096
CPPED1	0.7799023	0.0390932
TM4SF18	-1.580342	0.0391104
TMEM145	-0.836695	0.0392805
LOC506989	-1.629885	0.0393369
ITPKC	-0.838311	0.0394595
LOC509006	0.7932315	0.0394595
SCIN	-3.712522	0.0394595
TNIP3	-0.800023	0.0396435
RASD2	0.9048293	0.0398265
EOGT	-1.329989	0.0399619
ENSBTAG00000038702	-1.446178	0.0404095
TCN1	-2.311875	0.0404249
AKAP12	-1.549248	0.0405383
PCYOX1	-1.463339	0.0406058
APP	-0.766193	0.0406412
VDR	-1.181452	0.0406991
FBXL20	-0.933309	0.0413203
NECTIN2	-0.742823	0.0414462
AAAS	0.8500152	0.0414935
CCDC82	0.7570821	0.0414935
LITAF	-0.809276	0.041842
UBE2W	-0.883266	0.0419969
CALM3	0.7094564	0.0420622
B3GNT9	-1.224037	0.0420622

UNKL	-0.906148	0.0423593
CASD1	-1.07342	0.0427089
PAICS	0.8181306	0.0428539
PDCD1LG2	-1.631109	0.0428539
ATOX1	0.7624056	0.0428545
COLGALT1	0.7252252	0.0428832
PGS1	-0.74929	0.0429098
ENSBTAG00000052369	-1.418956	0.0430545
SCLY	-0.785074	0.0434716
ENSBTAG00000018137	-1.518297	0.0440637
C3	-1.285738	0.0440637
DEPTOR	-1.729383	0.0444678
PRXL2C	-0.990717	0.0447327
GALNT12	-1.327095	0.0448383
CA12	-1.100104	0.044921
CSTB	-0.76009	0.0451772
DCAF4	-0.830743	0.0452091
TSTD3	-1.086925	0.0453446
CCDC71L	-0.973754	0.0454647
SYCP3	-1.248185	0.0455562
ENSBTAG00000054738	-1.335163	0.0456799
GPX3	-1.038641	0.0458165
ACTRT3	-1.020587	0.0459501
TASP1	-1.201104	0.0459501
ENSBTAG00000012087	-1.104169	0.0462878
SCFD2	-0.786275	0.046554
BORA	0.8107434	0.0467866
CCDC152	-2.926149	0.0469728
ZKSCAN5	-0.775212	0.0469728
BOLA-DQB	-2.419818	0.0470809
ESPNL	-1.010767	0.0472391
PRKCA	-0.938479	0.0473302
TTF1	-0.78654	0.0473302
ENSBTAG00000050822	-0.952847	0.0478183
MVK	0.8682058	0.0478423
SLC25A27	-1.436335	0.0482428
F11R	-1.4883	0.0483437
TMEM134	-0.922506	0.0484396
ABCA3	-0.688077	0.0487402
ACHE	-1.542535	0.048963
TRAF1	-1.035659	0.0491194
WDR59	-0.809607	0.0491194
MIR147	-0.90706	0.0499736
AOX1	8.0736142	5.90E-39
LOC539009	6.4834585	3.49E-30
RFLNA	5.1023401	8.02E-29
ENSBTAG00000020684	3.6461942	1.40E-25
LOC515676	4.6484029	7.23E-24
PCLAF	3.960019	9.95E-23
STMN1	3.9644753	1.49E-22

UHRF1	3.8106615	2.14E-22
E2F8	3.9505451	1.52E-21
BUB1	3.2880732	1.66E-21
BIRC5	2.9463599	3.30E-20
CDCA8	3.3413042	1.59E-19
ESPL1	3.2677882	2.64E-19
TOP2A	3.5600431	2.64E-19
MKI67	3.8862232	2.90E-19
KIF15	3.5899281	2.26E-18
NCAPH	3.1194589	3.92E-18
CDK1	3.4933929	4.03E-18
FOXM1	3.4473201	4.10E-18
AURKB	3.0128297	2.72E-17
TCF19	3.6506609	3.78E-17
TAGAP	3.154507	1.06E-16
KIF11	3.1770253	1.63E-16
CDKN3	3.925759	1.81E-16
RRM2	3.4939665	2.55E-16
CLSPN	3.4278754	3.36E-16
ENSBTAG00000038893	6.1869075	4.63E-16
ENSBTAG00000034871	3.8075505	4.71E-16
KIFC1	2.9993235	4.93E-16
CREB3L3	7.9559231	4.93E-16
CDKN2C	3.7574368	5.04E-16
ASF1B	2.6585767	5.16E-16
CCL8	4.6511874	7.54E-16
RAD51AP1	3.2494644	8.58E-16
IQGAP3	3.621184	9.15E-16
KNTC1	3.0143974	1.93E-15
LMNB1	2.6137913	2.04E-15
ENSBTAG00000053827	4.6243815	5.48E-15
CCR6	3.6543283	5.82E-15
CCNA2	3.0272301	1.12E-14
DLGAP5	3.2333467	1.46E-14
CENPF	3.467364	1.57E-14
POLE	3.3399522	2.10E-14
HJURP	3.178936	2.24E-14
ENSBTAG00000049573	8.3576258	2.71E-14
DCLK2	6.4268741	2.95E-14
SHCBP1	3.1892827	2.99E-14
KIF18B	3.428348	3.72E-14
CDCA2	2.9968504	4.07E-14
KNL1	3.3575349	4.60E-14
MSR1	3.0565106	4.83E-14
CEP55	3.1954699	4.83E-14
KIF20A	2.9707096	5.52E-14
SAPCD2	3.1519553	5.63E-14
SLC35A1	2.1783064	5.75E-14
UBE2C	2.5847999	6.52E-14
NCAPG	2.6624057	8.84E-14

ENSBTAG00000039413	2.6307863	9.10E-14
CIT	3.3310802	1.45E-13
MYBL2	2.676974	1.51E-13
RAD51	2.6203729	1.62E-13
CCNB2	2.6425098	2.15E-13
ENSBTAG00000048514	5.2001417	2.61E-13
ASPM	2.9369147	2.67E-13
BRCA1	2.4467975	2.72E-13
CENPT	2.9593899	2.85E-13
KIF22	3.14034	3.76E-13
TPX2	2.6257276	6.13E-13
SMC2	2.6765017	6.35E-13
ST6GALNAC5	6.6404384	1.71E-12
E2F2	3.8463937	2.17E-12
PRC1	2.8834241	2.24E-12
MCM4	2.0855682	2.74E-12
SPAG5	3.1278555	6.50E-12
BUB1B	2.9524895	6.86E-12
MIS18BP1	3.1404425	7.70E-12
NUSAP1	3.2257539	8.78E-12
TICRR	3.070616	1.08E-11
ENSBTAG00000040564	4.407197	1.24E-11
ENSBTAG00000027412	2.8937893	1.30E-11
NEK2	3.1785256	1.35E-11
AURKA	2.3569318	1.50E-11
DEPDC1B	3.6389404	1.50E-11
CENPE	2.7475112	1.52E-11
PRR11	3.6316732	1.67E-11
ENSBTAG00000032217	2.9141828	1.70E-11
CARD11	4.4640122	2.00E-11
HASPIN	2.569268	2.10E-11
CKS2	2.3861183	2.22E-11
FABP5	2.6328585	2.31E-11
CCNB1	2.7510354	2.32E-11
CDCA5	3.2144757	2.47E-11
ARHGEF39	2.2910574	2.95E-11
NDC80	3.2821759	3.37E-11
LOC526163	3.9399976	3.87E-11
CHAF1A	2.1345311	3.90E-11
CKAP2L	2.4081419	4.07E-11
SGO1	2.5005301	5.41E-11
NPL	2.3202574	7.25E-11
CDC25B	2.0181451	7.86E-11
PTER	2.166674	9.52E-11
MCM2	2.5160308	9.66E-11
SPDL1	2.3945702	9.71E-11
HELLS	2.4632023	9.93E-11
FAM83D	2.9089016	1.02E-10
CCNF	1.9797545	1.15E-10
KIF23	2.2200553	1.16E-10

RBL1	2.7656409	1.20E-10
CKAP2	2.2733629	1.41E-10
CHTF18	2.7808982	1.52E-10
QPCT	2.7492349	1.77E-10
CCL2	2.9046933	1.78E-10
MASTL	2.8022178	1.84E-10
RACGAP1	2.4168555	1.87E-10
ENSBTAG00000053991	4.4038106	2.01E-10
CDT1	2.8751272	2.04E-10
ENSBTAG00000049212	4.5244107	2.15E-10
MTFR2	2.9031041	2.34E-10
FBXO5	2.9949718	3.45E-10
SLC25A35	2.7152967	3.46E-10
PCNA	2.2501119	3.50E-10
PARPBP	2.8521191	3.61E-10
KIR2DS1	4.5935824	3.73E-10
NUF2	2.6414619	4.17E-10
E2F1	2.4691453	4.22E-10
HGF	3.669009	6.04E-10
ARHGAP11A	3.0952492	7.45E-10
PBK	2.6299667	8.61E-10
TTK	2.8277945	8.77E-10
MCM7	1.9490147	1.13E-09
GGCT	2.2196195	1.19E-09
SPP1	3.4421468	1.20E-09
TRAIP	3.0612605	1.36E-09
SPC25	2.719752	1.40E-09
CDCA3	2.2738216	1.46E-09
RAD54L	2.2344086	1.74E-09
TREM2	2.2881155	1.98E-09
HAVCR2	2.9072097	2.21E-09
FANCI	1.9066672	2.36E-09
ORC1	2.5058023	2.55E-09
ERCC6L	2.4746478	2.64E-09
ENSBTAG00000007296	3.8810788	3.19E-09
KIF4A	2.7727515	3.25E-09
ENSBTAG00000026909	3.337352	3.28E-09
NSD2	1.8261986	3.47E-09
CKS1B	2.0449711	4.48E-09
TROAP	2.7675108	4.48E-09
LBH	2.435449	4.68E-09
MELK	1.9358235	4.77E-09
SDR42E2	3.8778728	5.71E-09
KIF2C	2.6904412	5.95E-09
RNASEH2A	2.2444241	7.25E-09
OIP5	2.8395564	7.35E-09
RECQL4	2.7349215	7.94E-09
TK1	1.9203057	9.41E-09
FANCD2	2.076954	9.98E-09
ARHGAP33	3.4574749	1.02E-08

ENSBTAG00000045689	2.0005831	1.03E-08
NCAPG2	2.282037	1.03E-08
RHNO1	2.0183506	1.06E-08
HMMR	2.0804998	1.10E-08
SMC4	1.8927517	1.28E-08
MCM3	2.3894996	1.39E-08
ESCO2	2.7204743	1.48E-08
ENSBTAG00000002002	2.0387051	1.67E-08
MCM10	1.9233	1.99E-08
ENSBTAG00000049207	4.1140586	2.75E-08
KPNA2	1.7012182	3.09E-08
XG	2.0571358	3.19E-08
LIG1	1.997555	3.43E-08
ENSBTAG00000025283	2.9939687	3.44E-08
ANGPT2	3.0784231	3.54E-08
PLK4	2.3299406	4.92E-08
BRCA2	2.4179332	5.40E-08
CENPM	2.0957718	5.83E-08
ECT2	2.0680594	5.97E-08
TRIP13	2.0524898	6.44E-08
KIF14	3.1575037	6.45E-08
CDC20	2.3304605	6.64E-08
ENSBTAG00000024874	2.5662267	6.79E-08
CENPK	2.871886	7.05E-08
PMCH	3.2213835	9.37E-08
GPSM2	2.2487917	9.56E-08
PLK1	2.0813105	9.65E-08
HAPLN4	4.1446329	1.05E-07
GMNN	2.0189646	1.06E-07
MCM5	2.1085195	1.07E-07
EZH2	1.6880776	1.17E-07
POLD1	1.9141124	1.22E-07
STIL	2.3142544	1.39E-07
CCHCR1	2.0242704	1.57E-07
ZNF367	2.5414709	1.57E-07
ADA2	2.784089	1.61E-07
SYNE2	2.4750653	2.02E-07
LRRC1	3.6798138	2.27E-07
CENPW	2.4628235	2.45E-07
FEN1	1.6536611	2.68E-07
DTL	2.3401407	2.79E-07
MCM6	1.4967196	2.91E-07
ENSBTAG00000053097	1.7929346	2.99E-07
GINS2	2.2696233	3.24E-07
DHFR	1.7765466	3.46E-07
MGME1	1.8875439	3.53E-07
CENPU	2.2264836	4.55E-07
ENSBTAG00000027075	2.2451521	4.55E-07
H2AZ1	1.5753987	4.62E-07
ENSBTAG00000040367	3.7131614	4.64E-07

BRIP1	2.6702763	5.20E-07
HACD4	2.7879194	5.22E-07
KNSTRN	1.760388	5.27E-07
TMEM47	2.416992	5.27E-07
RFC4	1.9410584	6.03E-07
GUSB	1.4127706	6.08E-07
ABCG2	3.3922123	6.65E-07
TMPO	2.071551	6.65E-07
LMNB2	1.5493046	6.93E-07
MIS18A	2.5814767	7.06E-07
DNA2	2.4541011	7.23E-07
POLQ	2.4971221	8.89E-07
LOC504858	2.420891	8.98E-07
HMGB2	1.4498957	9.04E-07
SKA1	2.2294955	9.09E-07
ENSBTAG00000047029	2.4883206	9.58E-07
CDC25C	2.6389807	1.05E-06
POC1A	1.7133023	1.07E-06
DSN1	1.7007771	1.07E-06
ENSBTAG00000048616	2.3933115	1.12E-06
ENSBTAG00000022275	2.0429484	1.15E-06
LOC529196	2.0326408	1.15E-06
CCNB3	2.1404423	1.28E-06
ENPP1	2.2363209	1.30E-06
FAM72A	2.7827301	1.90E-06
CENPA	2.0514519	2.00E-06
EPB41L3	2.098179	2.15E-06
ENSBTAG00000031242	2.1519383	2.16E-06
CENPS	1.7622389	2.55E-06
LYZ2	3.0861362	3.09E-06
CLEC3B	2.5891103	3.51E-06
F13A1	2.5147581	3.66E-06
FIGNL1	1.8848791	3.86E-06
NCAPD3	1.6901924	4.02E-06
CENPN	2.5135537	4.64E-06
CCND2	1.3341536	4.68E-06
SKA2	1.7049026	4.99E-06
MYBL1	2.6657607	5.64E-06
CCNE1	1.6561627	6.74E-06
ATAD5	1.7474954	6.88E-06
MS4A8	2.4944769	7.06E-06
SLC8A1	3.0530979	7.38E-06
GINS4	2.0782729	8.09E-06
PTTG1	2.3319426	8.33E-06
PARM1	1.5163014	8.99E-06
GTSE1	1.3704505	9.47E-06
GINS1	1.527187	9.81E-06
CRYM	4.0777458	1.08E-05
ANLN	1.8238743	1.14E-05
MMS22L	2.178312	1.17E-05

KIF24	1.6749013	1.21E-05
SORD	1.7032869	1.25E-05
DNAJC9	1.8531996	1.39E-05
EPB41L2	1.6321741	1.39E-05
SUV39H1	1.3126672	1.44E-05
PIF1	3.1803997	1.54E-05
ENSBTAG00000053898	1.571417	1.56E-05
POLA1	1.4051354	1.63E-05
ENSBTAG00000022715	3.3719983	1.69E-05
RPA2	1.5221476	1.70E-05
CEP43	1.3927802	1.70E-05
PHF19	1.3535742	2.00E-05
MEF2C	1.8729104	2.23E-05
STC1	4.2850276	2.25E-05
TUBB4B	1.4168153	2.32E-05
DACH1	2.5081071	2.33E-05
CEP72	2.100035	2.34E-05
CR2	4.4176007	2.43E-05
ZNF385A	1.3002624	2.51E-05
FOS	2.4864239	2.76E-05
CPT2	1.2352915	2.78E-05
TUBB2A	1.2881091	3.04E-05
LOC533307	3.0392266	3.04E-05
DPYSL2	1.694419	3.17E-05
KIF18A	2.2979538	3.31E-05
RRM1	1.3968944	3.77E-05
RNASET2	1.2083308	4.12E-05
CITED4	1.5901917	4.15E-05
CDC45	1.6680399	4.26E-05
COL5A3	2.8489178	4.28E-05
CENPL	1.7516757	4.32E-05
FAM20C	1.33793	4.43E-05
THEM6	2.6709817	4.53E-05
SLC37A2	1.8243326	4.58E-05
PPP1R9A	2.1675115	4.81E-05
ENSBTAG00000049323	1.5702954	5.13E-05
LOC781146	4.0399908	5.29E-05
ATAD2	2.2528273	5.43E-05
UNG	1.1970665	6.29E-05
KLHL3	2.4624258	6.53E-05
KIF20B	1.6766209	6.77E-05
SPC24	2.0145477	7.03E-05
EXO1	2.2739715	7.99E-05
LOC509972	2.8751132	8.51E-05
SPIRE2	1.312995	8.80E-05
HIRIP3	1.3763048	8.88E-05
WDR76	1.3394458	9.04E-05
WDHD1	1.3685768	9.09E-05
PPBP	2.0966446	9.24E-05
CIP2A	1.722728	9.41E-05

CDCA7	1.8619193	9.41E-05
PSRC1	1.7021611	9.41E-05
TGM3	3.115078	9.45E-05
MAP4K1	2.2678305	9.60E-05
BARD1	2.1723606	9.60E-05
POLE2	2.181981	9.61E-05
CKAP5	1.2046465	0.0001008
ENSBTAG00000046383	1.7894099	0.0001024
KCNE3	2.6006259	0.000104
PARP1	1.1703654	0.0001109
RMI2	1.5599652	0.0001262
PRIM1	1.5658949	0.0001264
CDKN2D	1.8399342	0.0001335
ENSBTAG00000003769	1.9977272	0.0001603
PARVB	1.9018888	0.0001655
KCNC4	1.8474041	0.0001688
CDC23	1.2654728	0.0001768
TACC3	1.1482058	0.0001806
NEIL3	1.9521981	0.0001832
NSL1	1.6649677	0.0001861
DBF4	1.5205678	0.0001912
DUT	1.8660978	0.0002054
CCL24	2.9955309	0.0002059
HNMT	1.1812194	0.0002072
TRPC6	2.4952128	0.0002308
DTYMK	1.3685616	0.0002337
GPR19	2.6048194	0.0002397
LOC511229	1.7959092	0.000258
NASP	1.2246431	0.0002656
MSH2	1.2315496	0.0002873
CENPX	1.2399526	0.0002906
IFT80	1.6628516	0.0002984
MTHFD1	1.3937016	0.000305
PALD1	2.3695816	0.0003371
PAK6	2.5600591	0.0003521
FXYD6	1.7412222	0.0003533
NDC1	1.886934	0.0004166
NRM	1.408633	0.0004166
ENSBTAG00000014988	2.8099985	0.000425
ENSBTAG00000050015	1.9905785	0.0004279
CEP152	1.51342	0.0004292
CTSL	4.7619999	0.0004297
CALY	2.3991413	0.000445
RPA1	1.1025341	0.000445
RGS1	1.7091296	0.0004853
APOLD1	1.9396334	0.0004927
ENSBTAG00000034185	1.2806365	0.0005046
NCAPD2	1.1123727	0.0005231
RAD18	1.4390622	0.0005335
MND1	2.1388506	0.0005478

SH2D3C	2.0066297	0.0005521
E2F7	2.1265281	0.0005667
INCENP	1.6529829	0.0005673
TP73	2.3194461	0.0005835
MKNK1	1.1538228	0.0005853
USP1	1.1874934	0.0006
POLH	1.296585	0.0006126
LOC788425	2.7233592	0.0006515
DYSF	1.7902552	0.0006701
GNG11	2.0064492	0.00068
CENPI	1.3708712	0.0006905
ENSBTAG00000046857	2.3287659	0.0006937
LOC101903064	2.8123247	0.0007018
DCK	1.4833275	0.0007154
LRR1	1.680206	0.0007219
CHEK1	1.4623787	0.0007309
CDC6	1.3195809	0.0007532
POLA2	1.1383595	0.0007812
CDC25A	1.5386651	0.0007824
PAM	1.1285743	0.0008641
EGR3	3.106683	0.000927
TUBB2B	1.3645782	0.0009428
PRAG1	1.8636005	0.0009499
MYO10	1.7870952	0.0009992
DEFB7	1.2998106	0.0010139
TONSL	1.2182332	0.0010664
TOPBP1	1.094077	0.001093
MPHOSPH9	1.1398556	0.0011112
ASL	1.1961861	0.0011226
ZWINT	1.3745658	0.0011282
ENSBTAG00000001858	2.0802465	0.0012171
CCNE2	1.6175578	0.0012757
RCC1	1.1489077	0.0013325
CORO7	1.0949616	0.0013514
NEMP1	1.5521951	0.0013781
TUBB	1.1210956	0.0013781
SLC2A4RG	1.1920892	0.001425
PBX3	1.1278662	0.0015394
LOC101904667	1.0654325	0.0015819
AREG	1.5148031	0.0015933
SEPTIN10	1.6064922	0.0016945
ENSBTAG00000051519	1.5366375	0.0017201
FKBP5	1.4702161	0.0018004
NMRAL1	1.0969079	0.0018617
RARS2	1.0989753	0.0019527
PROCR	1.8302768	0.0019527
C24H18orf54	2.0416999	0.0019776
S100A5	1.955598	0.0022297
ENSBTAG00000002290	1.3819623	0.0022707
RDM1	1.8948443	0.0023101

ANG2	2.1491484	0.0023186
SGO2	1.1032722	0.0023189
LPXN	1.4112472	0.0023329
DOCK8	1.1410268	0.0023349
UBE2T	1.1940526	0.0023481
CCDC50	1.0476023	0.002462
ZGRF1	1.2747853	0.0025298
OPRL1	1.8958989	0.0025662
MYH15	1.769956	0.0026343
KLHL5	1.0001062	0.0027588
PDGFB	2.3135322	0.0027592
ADK	1.1382961	0.0027691
CFAP20DC	1.6924625	0.0027725
DMKN	2.5409688	0.0027725
SACS	1.2940374	0.0029287
MFNG	1.4693948	0.0031143
ARHGAP18	1.5781757	0.0031439
ARHGAP15	1.8948833	0.0033576
GINS3	1.1840795	0.0033716
ENSBTAG00000049995	2.217719	0.0035514
LOC112443216	1.6551937	0.0035667
CYP3A5	1.6691144	0.0035667
MARCO	1.6069919	0.0036997
UBE2S	1.0642741	0.003796
SGPL1	1.0209472	0.0039085
CHEK2	1.1287157	0.0040505
HROB	1.498021	0.0044318
LOC782688	1.5956581	0.004439
CBX5	1.3159743	0.0046489
SKA3	1.2768667	0.0048542
FCER2	1.4730016	0.0049555
GJC1	2.2523895	0.0049791
BFSP2	1.5074465	0.0050017
DHCR24	1.124859	0.0050017
HYLS1	1.1028764	0.0055036
ENSBTAG00000035572	1.6874841	0.0058811
MMP13	1.7086851	0.0059909
HSF2BP	1.8926389	0.0061892
LOC514011	1.1310444	0.0064819
CENPQ	1.390032	0.0065718
LBR	1.4943927	0.0067931
ENSBTAG00000007093	1.5443903	0.0068792
XRCC1	1.0308536	0.0069246
LOC101905222	2.1191247	0.0070347
RAET1G	1.9216204	0.0073211
PRIM2	1.2798384	0.0073329
HAUS4	1.0106224	0.0073422
MAP3K6	1.1583974	0.0073645
ANG	1.8942261	0.0074785
ENSBTAG00000034662	2.0778481	0.0075592

GDA	1.6605628	0.0075872
NMNAT2	1.657876	0.0076876
SFXN3	1.0464632	0.0077275
PADI4	1.2628693	0.0078124
CEP57	1.2240712	0.0078887
RASAL3	1.4299961	0.0079369
TST	1.457948	0.0081652
SLAIN1	1.5757345	0.0083914
ENSBTAG00000050785	1.6063882	0.0084043
BANK1	1.0374081	0.0088896
FAM111B	1.019703	0.0088919
CD48	1.1855738	0.0089423
KLHL6	1.5770315	0.0090967
RPA3	1.1045522	0.0092904
ENSBTAG00000015149	1.2057518	0.0096652
LOC510798	1.0066586	0.0096664
LAMB1	1.2115841	0.0100315
FOLH1B	1.6646092	0.0101996
PARD6G	1.4334893	0.0102406
ETV5	1.0852209	0.010397
QRICH2	1.8084002	0.0105691
ENSBTAG00000051299	1.6792995	0.0106368
FKBP9	1.0160767	0.0106371
ENSBTAG00000051402	1.2765366	0.0106479
IPP	1.2176773	0.0107091
PALB2	1.0987511	0.0109194
ATP2A3	1.5218577	0.0109903
GPR34	2.1891565	0.0113841
ARHGAP19	1.4782823	0.0114845
CMC2	1.0085246	0.0116986
TENT5B	1.1835753	0.0118749
P2RY11	1.640172	0.0119031
BRI3BP	1.4658182	0.0121683
ITGAM	1.3176526	0.0121683
ADGRF5	2.9316277	0.012192
SEH1L	1.0352349	0.01248
POT1	1.1518025	0.0125288
TREML1	2.1998117	0.0133939
C1QC	1.6800338	0.0134186
PMP22	1.2125762	0.0149641
ENSBTAG00000027426	1.6071335	0.0149641
CDCA7L	1.0752344	0.0149684
SLC9A9	1.1928601	0.0149684
DEFB13	2.2013885	0.0150308
SORL1	2.0162263	0.0153932
HSD17B11	1.3091972	0.015724
WNK2	1.5228096	0.0159119
CDR2	1.4969729	0.0159705
ENSBTAG00000048517	1.8301731	0.016051
SHMT1	1.695137	0.0170968

IL4I1	1.644552	0.0171505
MCM8	1.2707923	0.0177722
LIN9	1.2294999	0.0179268
RFESD	1.6350664	0.0180011
CRACR2A	1.2371157	0.0180011
ENSBTAG00000011636	1.0087676	0.0181735
LOC404051	2.0138829	0.0185542
OAF	1.7822954	0.0186407
ENSBTAG00000047637	1.4187135	0.0186743
PDE10A	1.4611983	0.0187697
LOC613822	1.2740232	0.0188385
LOC101902561	1.2743432	0.0191571
NXPH3	1.6692843	0.019738
SNAP25	1.5476565	0.0202653
TMEM69	1.1138969	0.0202792
RGS2	1.7237002	0.0202862
MYEF2	1.2667656	0.0219225
ENSBTAG00000021649	1.5159517	0.0221585
RAD54B	1.1798284	0.0221585
LOC784052	1.5300625	0.022437
COMTD1	1.2246318	0.022437
LOC107132247	1.489114	0.0232061
FAM107B	1.7422708	0.0236175
CENPO	1.1006138	0.0239372
LMO2	1.5527305	0.0246522
TMC8	1.2013385	0.0247436
LOC101906312	1.3274396	0.0247436
CDH26	2.1653731	0.0247467
ENSBTAG00000050608	2.7328703	0.0251721
ENSBTAG00000003743	1.809597	0.0260993
MAMLD1	1.1747804	0.026205
CENPP	1.3912997	0.026205
TMEM170B	1.3207731	0.026843
RGS16	1.3271998	0.026843
PKP4	1.0312663	0.0270178
ALOX5AP	1.3056763	0.0287504
AGAP2	1.4850086	0.0294726
IGF2BP3	1.0801037	0.0299233
CCDC88C	1.121435	0.0305452
MTBP	1.139501	0.0305473
VSIG4	1.1750413	0.0318909
FNDC10	1.438216	0.0320145
ENSBTAG00000052532	1.1852039	0.0322883
USHBP1	1.2331641	0.0324735
EFR3B	1.2249575	0.0344655
RBM38	1.1197572	0.0359116
SIVA1	1.1255867	0.0364661
UNC93A	2.1118788	0.0399619
THRB	1.4541823	0.0402314
CGAS	1.1898146	0.0413203

ECHDC2	2.3500715	0.0428539
ATP10A	1.4593021	0.044004
ENSBTAG00000052184	1.2336381	0.0451772
LMAN2L	1.3072469	0.046554
ITGA6	1.247198	0.0469728
NUDT1	1.0049672	0.0469957
CD300LF	1.4758647	0.0476016
ENSBTAG00000054407	1.6627127	0.0484108
