# Supplementary material for: Lipopolysaccharide triggers different transcriptional signatures in taurine and indicine cattle macrophages: Reactive oxygen species and potential outcomes to the development of immune response to infections
Source: PLoS One. 2020 Nov 6;15(11):e0241861. doi: 10.1371/journal.pone.0241861 (PMC7647108; doi:10.1371/journal.pone.0241861)
Supplement: S6 Table — DEG enrichment analysis performed by DAVID with data from unstimulated MDMs from Holstein versus Gir animals, showing biological processes and associated genes with statistical significance (P value and FDR). The “Count” column shows the number of enriched genes for each process. (PDF) [file pone.0241861.s008.pdf]

Term	Count	PValue	FDR	Genes
Immune response	8	0,001	1,990	*BOLA-DQA2, CCR5, BOLA-DQA5, OAS1Z, TGFBR3, LOC529196, BOLA-DQB, BMPR1A*
Antigen processing and presentation of peptide or polysaccharide antigen via MHC class II	3	0,008	11,380	*BOLA-DQA2, BOLA-DQA5, BOLA-DQB*
Citrate transport	2	0,015	20,007	*SLC13A5, SLC13A3*
Positive regulation of TOR signaling	3	0,018	23,376	*LOC614531, RRAGA, LOC539009*
Regulation of autophagy	3	0,025	30,807	*LOC614531, RRAGA, LOC539009*
Base-excision repair, gap-filling	2	0,031	36,013	*POLD1, POLE*
Sensory perception of sound	4	0,034	38,733	*TUB, ATP8B1, CRYM, CDH23*
Cellular response to starvation	3	0,037	41,399	*LOC614531, RRAGA, LOC539009*
Nucleotide-excision repair, DNA gap filling	2	0,038	42,772	*POLD1, POLE*
G1/S transition of mitotic cell cycle	3	0,038	42,893	*POLE, CDKN3, CDCA5*
Stem cell population maintenance	3	0,038	42,893	*PHF19, PADI4, BMPR1A*
Cellular response to amino acid stimulus	3	0,045	48,760	*LOC614531, RRAGA, LOC539009*
