# Supplementary material for: Lipopolysaccharide triggers different transcriptional signatures in taurine and indicine cattle macrophages: Reactive oxygen species and potential outcomes to the development of immune response to infections
Source: PLoS One. 2020 Nov 6;15(11):e0241861. doi: 10.1371/journal.pone.0241861 (PMC7647108; doi:10.1371/journal.pone.0241861)
Supplement: S7 Table — DEG enrichment analysis performed by DAVID with data from unstimulated versus LPS treated MDMs from Holstein breed, showing biological processes and associated genes with statistical significance (P value and FDR). The “Count” column shows the number of enriched genes for each process. (PDF) [file pone.0241861.s009.pdf]

Term	Count	PValue	FDR	Genes
Immune response	9	4,8E-05	0,069	*CSF3, BOLA-DMA, CXCL3, CXCL2, IL1B, NRROS, GRO1, IL1A*
Inflammatory response	9	4,8E-05	0,069	*MEFV, CXCL3, CXCL2, AXL, NRROS, GRO1, CCL5, CCL4*
Chemokine-mediated signaling pathway	5	1,4E-04	0,209	*CXCL3, CXCL2, CCL5, CCL4*
Cell chemotaxis	5	1,9E-04	0,270	*CXCL3, CXCL2, SAA3, GRO1*
Metabolic process	6	2,4E-04	0,348	*LOC615514, GSTM1, GSTM2, ASPA, ACSF2*
Cell maturation	4	9,5E-04	1,371	*GATA3, POU2F2, PPARG, AXL*
Positive regulation of inflammatory response	4	0,002	3,015	*S100A8, S100A9, CCL5, CCL4*
Neutrophil chemotaxis	4	0,003	4,144	*S100A8, S100A9, CCL5, CCL4*
Superoxide metabolic process	3	0,004	5,184	*NRROS, SOD2*
Cellular response to tumor necrosis factor	4	0,005	7,398	*GATA3, ZC3H12A, CCL5, CCL4*
Positive regulation of protein kinase B signaling	4	0,006	7,747	*CSF3, GATA3, AXL, ITSN1*
Positive regulation of neutrophil chemotaxis	3	0,006	8,188	*CXCL3, CXCL2*
Fever generation	2	0,012	16,305	*IL1B, IL1A*
Age-dependent response to reactive oxygen species	2	0,012	16,305	*SOD2*
Neutrophil aggregation	2	0,012	16,305	*S100A8, S100A9*
Positive regulation of peptide secretion	2	0,012	16,305	*S100A8, S100A9*
Response to lipopolysaccharide	4	0,014	18,640	*IL23R, CXCL3, CXCL2*
Negative regulation of I-kappaB kinase/NF-kappaB signaling	3	0,016	21,077	*NLRP12, ZC3H12A, TNIP3*
Negative regulation of neuron apoptotic process	4	0,017	22,027	*MT2A, AXL, CNTFR, ITSN1*
Positive regulation of natural killer cell chemotaxis	2	0,024	29,953	*CCL5, CCL4*
Calcium ion transport	3	0,026	31,591	*RAMP3, CACNA1G, CCL5*
Transmembrane transport	4	0,029	35,260	*SLC13A5, BCL2A1, SLC37A2, GJB2*
Activation of cysteine-type endopeptidase activity involved in apoptotic process	3	0,031	36,391	*S100A8, PPARG, S100A9*
Cellular response to interleukin-1	3	0,033	38,787	*ZC3H12A, CCL5, CCL4*
Transforming growth factor beta receptor signaling pathway	3	0,040	44,708	*FOS, ITGB5, CDH5*
Leukocyte migration involved in inflammatory response	2	0,042	46,370	*S100A8, S100A9*
Cellular response to interferon-alpha	2	0,042	46,370	*GATA3, AXL*
Negative regulation of cytokine production involved in inflammatory response	2	0,042	46,370	*MEFV, ZC3H12A*
