# Supplementary material for: Lipopolysaccharide triggers different transcriptional signatures in taurine and indicine cattle macrophages: Reactive oxygen species and potential outcomes to the development of immune response to infections
Source: PLoS One. 2020 Nov 6;15(11):e0241861. doi: 10.1371/journal.pone.0241861 (PMC7647108; doi:10.1371/journal.pone.0241861)
Supplement: S8 Table — DEG enrichment analysis performed by DAVID with data from unstimulated versus LPS treated MDMs from Gir breed, showing biological processes and associated genes with statistical significance (P value and FDR). The “Count” column shows the number of enriched genes for each process. (PDF) [file pone.0241861.s010.pdf]

Term	Count	PValue	FDR	Genes
Chemokine-mediated signaling pathway	12	1,32E-09	2,20E-06	*CCL24, CCL3, CCL2, CXCL5, CXCR4, CXCL3, CXCL2, CCL8, CXCL8, CCL5, CCL4*
Cellular response to tumor necrosis factor	13	2,45E-09	4,08E-06	*PID1, IL6, CCL3, CCL2, CXCL8, CCL8, ANKRD1, CCL5, CCL4, DCSTAMP, CCL24, OCSTAMP, GATA3*
Cell chemotaxis	12	2,84E-09	4,73E-06	*CCL24, C3AR1, CCL2, CXCL5, SAA2, CXCL3, CXCL2, SAA3, HBEGF, GRO1, M-SAA3.2*
Inflammatory response	20	1,58E-07	2,62E-04	*C3AR1, CCL3, CCL2, CXCL5, PTGS2, LYN, CXCL3, CSF1, CXCL2, AXL, CCL8, CXCL8, GRO1, CCL5, CCL4, CCL24, LAT, MEFV, CLEC7A*
Positive regulation of inflammatory response	10	1,84E-07	3,07E-04	*WNT5A, CCL24, CCL3, CCL2, S100A8, S100A9, CCL8, IL33, CCL5, CCL4*
Positive regulation of angiogenesis	13	2,17E-07	3,61E-04	*WNT5A, CCL24, C3AR1, FLT1, CYP1B1, F3, SERPINE1, TGFBR2, CCBE1, VEGFA, CXCL8, FGF1, IL1A*
Neutrophil chemotaxis	10	5,13E-07	8,54E-04	*CCL24, CCL3, CCL2, S100A8, S100A9, CCL8, CXCL8, JAML, CCL5, CCL4*
Cellular response to interleukin-1	10	5,13E-07	8,54E-04	*CCL24, CCL3, IL6, CCL2, PTGS2, CCL8, CXCL8, ANKRD1, CCL5, CCL4*
Immune response	19	7,48E-07	0,001	*CSF3, CCL3, CCL2, CXCL5, BLA-DQB, CXCL3, CXCL2, CXCL8, GRO1, TINAGL1, CCL24, LAT, BOLA-DMA, BOLA-DMB, IL1B, BOLA-DRA, IL1A, BLNK*
Angiogenesis	15	9,47E-07	0,002	*CYP1B1, ANG2, MFGE8, EPHB3, MMP2, PTEN, XBP1, ANG, PLXDC1, VEGFA, TGFBI, SERPINE1, PLCD1, FGF1, FN1*
Protein kinase B signaling	7	1,39E-05	0,023	*TYRO3, CCL3, CCL2, AXL, CCL5, PTEN, GAS6*
Collagen fibril organization	7	2,71E-05	0,045	*CYP1B1, COL3A1, COL1A2, COL1A1, LOXL2, COL5A2, SERPINH1*
Monocyte chemotaxis	7	2,71E-05	0,045	*CCL24, CCL3, CCL2, FLT1, CCL8, CCL5, CCL4*
Lymphocyte chemotaxis	6	8,10E-05	0,135	*CCL24, CCL3, CCL2, CCL8, CCL5, CCL4*
Cell adhesion	14	8,13E-05	0,135	*CTNNAL1, PTPRF, ADGRE5, TNC, ITGB5, MFGE8, ITGA3, CERCAM, TINAGL1, TGFBI, COL12A1, GPNMB, MYH10, FN1*
Cellular response to interferon-gamma	7	9,82E-05	0,163	*WNT5A, CCL24, CCL3, CCL2, CCL8, CCL5, CCL4*
Cellular response to amino acid stimulus	7	3,09E-04	0,512	*XBP1, COL3A1, COL1A2, COL6A1, COL1A1, MMP2, COL5A2*
Cellular response to interferon-alpha	4	3,10E-04	0,514	*IFIT3, GATA3, AXL, GAS6*
Positive regulation of endothelial cell migration	6	3,81E-04	0,632	*WNT5A, GATA3, VEGFA, CCBE1, SPARC, FGF1*
Acute-phase response	5	4,78E-04	0,793	*IL6, SAA2, SAA3, M-SAA3.2, FN1*
Positive regulation of neutrophil chemotaxis	5	5,96E-04	0,987	*C3AR1, CXCL3, CXCL2, CXCL8*
Cellular response to lipopolysaccharide	8	6,85E-04	1,133	*WNT5A, IL6, XBP1, SERPINE1, AXL, CXCL8, ANKRD1, TNIP3*
Protein heterotrimerization	4	7,20E-04	1,191	*COL6A2, COL1A2, COL6A1, COL1A1*
Response to hypoxia	8	8,82E-04	1,457	*CXCR4, ANG, PLOD2, VEGFA, TH, EDN1, LOXL2, MMP2*
Positive regulation of smooth muscle cell proliferation	5	8,90E-04	1,470	*IL6, HMOX1, EDN1, HBEGF, CCL5*
Positive regulation of macrophage chemotaxis	4	0,001	1,671	*C3AR1, CCL2, CSF1, CCL5*
Positive regulation of vascular endothelial growth factor production	5	0,001	1,764	*C3AR1, CYP1B1, SULF2, CCBE1, IL1A*
Regulation of vascular endothelial growth factor production	3	0,001	2,180	*IL6, CCL2, NDRG2*
Negative regulation of ERK1 and ERK2 cascade	6	0,001	2,435	*LYN, NLRP12, NDRG2, TIMP3, PTEN, SPRY4*
Positive regulation of protein kinase B signaling	7	0,002	2,485	*CSF3, F3, GATA3, AXL, HBEGF, MST1R, GAS6*
Positive regulation of ERK1 and ERK2 cascade	10	0,002	2,942	*CCL24, GPR183, CCL3, CCL2, CCL8, CCL5, SLAMF1, PTEN, CCL4, GAS6*
Endodermal cell differentiation	5	0,002	3,348	*COL12A1, ITGB5, COL6A1, MMP2, FN1*
Negative regulation by host of viral transcription	4	0,002	3,761	*CCL3, HMOX1, CCL5, CCL4*
Positive regulation of natural killer cell chemotaxis	3	0,003	4,256	*CCL3, CCL5, CCL4*
Positive regulation of calcium ion transport	4	0,003	4,691	*CCL3, TRPC6, CCL5, CCL4*
RNA phosphodiester bond hydrolysis	3	0,004	6,904	*ANG, RNASE4, ANG2*
Cell-substrate junction assembly	3	0,004	6,904	*TNS1, ITGB3, FN1*
Positive regulation of protein kinase activity	5	0,004	7,156	*LAT, EREG, CSF1, GAS6, VLDLR*
Antigen processing and presentation of peptide or polysaccharide antigen via MHC class II	4	0,006	9,636	*BOLA-DMA, BLA-DQB, BOLA-DMB, BOLA-DRA*
Dendritic cell differentiation	3	0,006	10,050	*LYN, AXL, GAS6*
Secretion by cell	3	0,006	10,050	*TYRO3, SERPINE2, AXL*
Negative regulation of cell proliferation	12	0,007	10,496	*IFIT3, CYP27B1, CYP1B1, SERPINE2, EREG, GATA3, ROR2, FOXO4, KLF4, IL1A, KANK2, TES*
Positive regulation of fibroblast proliferation	5	0,007	10,772	*WNT5A, EREG, ITGB3, GAS6, FN1*
Endothelial cell migration	4	0,007	11,177	*CYP1B1, LOXL2, PTEN, STARD13*
Negative regulation of interferon-gamma production	4	0,007	11,177	*GATA3, AXL, IL33, GAS6*
Positive regulation of peptidyl-serine phosphorylation	6	0,008	11,881	*CSF3, WNT5A, PFN2, IL6, VEGFA, GAS6*
Positive regulation of immunoglobulin secretion	3	0,009	13,618	*IL6, XBP1, IL33*
Skin morphogenesis	3	0,009	13,618	*COL1A2, COL1A1, ERRFI1*
Eosinophil chemotaxis	3	0,009	13,618	*CCL24, CCL3, CCL5*
Cytokine-mediated signaling pathway	8	0,011	16,407	*IL6, BGN, EREG, F3, IL1B, CISH, IL1A, CHAD*

Calcium ion transport	5	0,011	16,542	*VDR, CCL3, CYP27B1, CCL8, CCL5*
Vagina development	3	0,012	17,531	*WNT5A, TYRO3, AXL*
Negative regulation of receptor binding	3	0,012	17,531	*PTPRF, HFE, ADAM15*
Integrin-mediated signaling pathway	6	0,012	18,360	*LAT, COL3A1, ITGB5, ITGA3, ITGB3, ADAM15*
Calcium-mediated signaling	4	0,012	18,459	*LAT, CCL3, CXCR4, EDN1*
Cell migration	8	0,013	19,278	*PTPRF, LYN, FSCN1, PTK7, ASAP3, EPHB3, PALLD, SDC2*
Canonical Wnt signaling pathway	6	0,015	21,619	*WNT5A, PTK7, FZD1, PTEN, KLF4, BCL9*
Cell-cell signaling	6	0,015	21,619	*CCL3, EREG, LVRN, CCL8, CCL5, GJB2*
Skin development	4	0,015	22,689	*COL3A1, ITGA3, COL5A2, ABCB6*
Positive regulation of cell division	4	0,015	22,689	*VEGFA, IL1B, FGF1, IL1A*
Exocytosis	5	0,016	23,597	*CCL3, CCL8, SYTL2, CCL5, MYH10*
Cellular response to cytokine stimulus	3	0,018	26,082	*CSF3, CXCR4, NLRP12*
Apoptotic cell clearance	3	0,018	26,082	*TYRO3, AXL, GAS6*
Positive regulation of cell migration	8	0,018	26,696	*DAB2, CCL3, FLT1, LYN, EDN1, ROR2, COL1A1, FGF1*
Positive regulation of endothelial cell proliferation	5	0,019	26,727	*WNT5A, CCL24, ANG, F3, VEGFA*
Lipopolysaccharide-mediated signaling pathway	4	0,021	29,593	*CCL3, CCL2, LYN, CCL5*
Cellular response to fibroblast growth factor stimulus	3	0,022	30,577	*CCL2, CXCL8, CCL5*
Positive regulation of transcription from RNA polymerase II promoter	21	0,022	31,471	*CSF3, PID1, WNT5A, IL6, CCL3, LMO2, EDN1, FSTL3, EHF, ANKRD1, IL33, FOXO4, VDR, XBP1, GATA3, CD81, VEGFA, CREB3L1, FGF1, IL1A, BCL9*
Blood coagulation	5	0,023	31,690	*F2RL2, SERPINE2, F3, PROS1, GAS6*
Negulation of cell shape	7	0,024	33,458	*CCL24, CCL3, CCL2, VEGFA, CDC42EP3, FN1, MYH10*
Collagen catabolic process	3	0,026	35,129	*MRC2, MMP2, MMP1*
Planar cell polarity pathway involved in neural tube closure	3	0,026	35,129	*WNT5A, PTK7, FZD1*
Decidualization	3	0,026	35,129	*VDR, CYP27B1, PTGS2*
Natural killer cell differentiation	3	0,026	35,129	*TYRO3, AXL, SLAMF1*
Negative regulation of tumor necrosis factor production	4	0,027	36,947	*AXL, GPNMB, SLAMF1, GAS6*
Cellular oxidant detoxification	4	0,027	36,947	*S100A8, PTGS2, NXN, S100A9*
Heart development	7	0,028	38,085	*ADAP2, GATA3, COL3A1, TH, TGFBR2, EDN1, ID3*
Response to lipopolysaccharide	6	0,031	41,213	*CYP27B1, CXCL5, CXCL3, CXCL2, CXCL8*
Positive regulation of gene expression	8	0,032	41,396	*ACTG2, CCL3, TNC, CSF1, HFE, ITGB3, GAS6, FN1*
Cellular response to organic cyclic compound	4	0,032	41,969	*CCL3, CCL2, CYP1B1, CCL5*
Activation of protein kinase B activity	3	0,034	44,177	*WNT5A, FGF1, GAS6*
Astrocyte development	3	0,034	44,177	*LAMB2, S100A8, S100A9*
Regulation of blood pressure	4	0,035	44,487	*PTGS2, EDN1, LVRN, COL1A2*
Regulation of cell proliferation	8	0,036	45,615	*CXCL5, PTGS2, CXCL3, TNC, CXCL2, CXCL8, PLCD1*
Positive regulation of protein phosphorylation	5	0,036	45,867	*PTGS2, VEGFA, FZD1, CLIP3, GAS6*
Negative regulation of apoptotic process	11	0,037	46,606	*IFIT3, WNT5A, IL2RB, DAB2, IL6, XBP1, VEGFA, SERPINB2, ASNS, PTEN*
Positive regulation of actin filament polymerization	4	0,037	46,996	*CSF3, CCL24, PFN2, CDC42EP3*
Response to toxic substance	4	0,037	46,996	*CCL3, CYP1B1, CCL5, CCL4*
Positive regulation of monocyte chemotaxis	3	0,039	48,576	*CCL2, SERPINE1, CCL5*
Superoxide metabolic process	3	0,039	48,576	*CBS, SOD2*
Wound healing	4	0,040	49,489	*WNT5A, COL3A1, PTK7, FN1*
Negative regulation of interleukin-1 secretion	2	0,042	50,966	*NLRP12, GAS6*
Fever generation	2	0,042	50,966	*IL1B, IL1A*
Ovulation cycle	2	0,042	50,966	*TYRO3, AXL*
Regulation of B cell apoptotic process	2	0,042	50,966	*LYN, PTEN*
Neutrophil aggregation	2	0,042	50,966	*S100A8, S100A9*
Age-dependent response to reactive oxygen species	2	0,042	50,966	*SOD2*
Positive regulation of peptide secretion	2	0,042	50,966	*S100A8, S100A9*
Negative regulation of dendritic cell apoptotic process	2	0,042	50,966	*AXL, GAS6*
Substrate adhesion-dependent cell spreading	4	0,043	51,956	*TYRO3, AXL, EPHB3, FN1*
Positive chemotaxis	3	0,044	52,838	*CCL3, SAA2, CCL5*
Positive regulation of mitotic nuclear division	3	0,044	52,838	*EREG, EDN1, IL1A*
Myeloid dendritic cell differentiation	3	0,044	52,838	*BATF3, TGFBR2, DCSTAMP*
Cellular calcium ion homeostasis	5	0,044	53,052	*VDR, CCL3, CCL8, STC1, CCL5*
