# Supplementary material for: Lipopolysaccharide triggers different transcriptional signatures in taurine and indicine cattle macrophages: Reactive oxygen species and potential outcomes to the development of immune response to infections
Source: PLoS One. 2020 Nov 6;15(11):e0241861. doi: 10.1371/journal.pone.0241861 (PMC7647108; doi:10.1371/journal.pone.0241861)
Supplement: S9 Table — DEG enrichment analysis performed by DAVID with data from LPS treated MDMs from Holstein versus Gir animals, showing biological processes and associated genes with statistical significance (P value and FDR). The “Count” column shows the number of enriched genes for each process. (PDF) [file pone.0241861.s011.pdf]

Term	Count	PValue	FDR	Genes
Cell division	45	1,75E-17	3,13E-14	*CKS1B, AURKA, PTTG1, FAM83D, SPC24, CCNE2, SPC25, NCAPH, VRK1, SEH1L, MIS18A, CDCA2, SKA3, SKA2, MASTL, SKA1, CDCA5, CCNA2, ASPM, BUB3, CDCA3, ERCC6L, KIF14, CDK1, CDC6, DSN1, CCNF, PSRC1, TPX2, CDC23, BIRC5, SPDL1, CDC25C, UBE2C, CDC25A, RBBP8, CCNB1, CCNB2, CCND3, SYCP3, CCND2, ZWINT, CKS2, UBE2S*
DNA replication initiation	15	2,72E-12	4,87E-09	*CCNE2, CCNE1, CDC6, CDC45, MCM7, GINS4, POLA1, TOPBP1, MCM2, POLA2, MCM10, MCM3, MCM4, MCM5, MCM6*
Chromosome segregation	18	2,89E-09	5,17E-06	*CENPN, KIF11, NEK3, NEK2, BIRC5, RCC1, KNSTRN, BRCA1, SPC25, OIP5, HJURP, MIS18A, CENPW, SKA3, SKA2, CENPT, SKA1, CDK5RAP2*
DNA replication	20	3,56E-09	6,38E-06	*RECQL4, TICRR, NASP, GINS3, POLE, RMI2, NFIX, POLA2, BRCA1, RPA3, RPA1, POLD3, RPA2, DNA2, MCM8, POLD1, RRM1, POLQ, CHAF1A, ORC1*
Mitotic nuclear division	27	4,41E-09	7,90E-06	*PTTG1, FAM83D, SPC24, SPC25, VRK1, MIS18A, CDCA2, SKA3, SKA2, MASTL, SKA1, CCNA2, ASPM, ERCC6L, CDK1, CENPN, CCNF, NUF2, GEM, CDC25C, CDC25A, RBBP8, CCNB2, PLK1, ZWINT, CENPW*
Mitotic sister chromatid segregation	11	4,92E-08	8,82E-05	*CDCA8, CEP57, PLK1, NEK2, SPAG5, DSN1, KIF18A, KIF18B, ESPL1, KNSTRN, BUB3*
Base-excision repair	13	2,29E-07	4,09E-04	*RECQL4, HMGB1, NEIL3, LIG1, XRCC1, HMGA1, RPA3, RPA1, RPA2, DNA2, MUTYH, POLQ, FEN1*
Mitotic cytokinesis	11	1,52E-06	2,72E-03	*KIF23, CKAP2, BBS4, PLK1, NUSAP1, ANLN, CEP55, STMN1, RACGAP1, MYH10, KIF20A*
Mitotic metaphase plate congression	12	2,98E-06	0,005	*KIF14, CCNB1, KIF2C, KIF22, KIFC1, CDCA8, SEH1L, PSRC1, KIF18A, CDC23, SPDL1, CDCA5*
Mitotic spindle organization	10	9,55E-06	0,017	*CCNB1, SPC25, STIL, PCNT, GPSM2, AURKA, NDC80, AURKB, STMN1, RCC1*
Microtubule-based movement	15	1,17E-05	0,021	*KIF14, KIF23, KIF22, KIFC1, KIF4A, KIF11, KIF24, KIF15, KIF18A, KIF18B, CENPE, KIF2C, KIF7, KIF20B, KIF20A*
DNA duplex unwinding	9	1,60E-05	0,029	*GINS1, RECQL4, DNA2, GINS2, GINS4, MCM3, POT1, MCM5, MCM6*
Cell adhesion	28	3,57E-05	0,064	*CTNNAL1, MPZL3, ACHE, TLN2, TNC, FERMT1, NINJ1, ITGAM, COL12A1, SPP1, FN1, PRKCA, ICAM1, F11R, HAPLN4, HAPLN3, OLR1, PTPRF, MFGE8, TINAGL1, CERCAM, ITGA9, LAMA4, FBLN5, PECAM1, TCAM1, PARVB, MYH10*
G2/M transition of mitotic cell cycle	10	4,29E-05	0,077	*NES, PLK1, FOXM1, KDM8, CALM3, BIRC5, CHEK1, MASTL, CHEK2, CDC25C*
Mitotic chromosome condensation	7	6,00E-05	0,107	*NCAPH, NCAPG, NUSAP1, SMC2, CDCA5, NCAPD3, SMC4*
Cerebral cortex development	11	9,36E-05	0,168	*KIF14, SMO, BBS4, HIF1A, CCDC85C, MCPH1, COL3A1, PTPRS, CDH2, TACC3, ASPM*
Cellular response to amino acid stimulus	12	1,20E-04	0,215	*LOC614531, SOCS1, COL3A1, PDGFRA, COL1A2, COL6A1, RRAGA, COL1A1, MMP2, COL5A2, NEURL1, LOC539009*
Positive regulation of cytokinesis	9	2,17E-04	0,388	*KIF23, KIF14, CDC6, PKP4, AURKB, RACGAP1, CIT, ECT2, CDC25B*
Regulation of attachment of spindle microtubules to kinetochore	5	2,76E-04	0,493	*NEK2, SPAG5, RACGAP1, KNSTRN, ECT2*
Double-strand break repair via homologous recombination	13	3,44E-04	0,614	*MMS22L, TONSL, BRCA2, RAD54L, BRCA1, RAD51, RPA3, RBBP8, RPA1, RPA2, MCM8, RAD54B, PALB2*
Microtubule depolymerization	6	4,52E-04	0,806	*KIF2C, KIF24, KIF18A, KIF18B, STMN1, NCKAP5L*
Collagen fibril organization	9	4,97E-04	0,886	*FMOD, LUM, COL3A1, COL1A2, LOX, COL1A1, LOXL2, SERPINH1, COL5A2*
Cell cycle	17	5,06E-04	0,903	*CKAP2, LIN9, NASP, RBL1, SUV39H1, MCM3, MCM5, BRCA1, MCM6, CCNE2, UHRF1, MCM8, MCM7, RGS2, CCND3, CCND2, CHAF1A*
Microtubule bundle formation	8	5,83E-04	1,039	*PRC1, PLK1, TPPP, PSRC1, MAP1B, CDK5RAP2, NCKAP5L, KIF20A*
DNA unwinding involved in DNA replication	5	6,09E-04	1,086	*MCM7, MCM2, TOP2A, MCM6, RAD51*
G1/S transition of mitotic cell cycle	10	0,001	2,007	*CCNE2, CCNE1, CDKN2C, CDKN2D, POLE, IQGAP3, RCC1, CDKN3, CDCA5, RBBP8*
Protein localization to kinetochore	5	0,001	2,046	*CDK1, MTBP, BUB1B, SPDL1, AURKB*
Homologous chromosome segregation	4	0,001	2,058	*PLK1, ESPL1, PTTG1*
Inflammatory response	28	0,001	2,300	*HMGB1, CCL2, PTGS2, C3, CXCL3, CSF1, CCL8, TLR4, NFKB2, MMP25, CCRL2, CCL24, S1PR3, SLC11A1, NRROS, NOS2, FAS, LOC510798, IRAK2, OLR1, AXL, LOC529196, GGT5, CCR5, PPBP, TBXA2R, PTAFR, IL36A*
DNA repair	18	0,001	2,325	*KIF22, NUDT1, POLH, USP1, CHEK1, PTTG1, BRCA1, PAXIP1, UHRF1, FANCI, FANCD2, PARPBP, RAD18, UBE2W, CHAF1A, UBE2T, RDM1*
Chromosome organization	6	0,002	2,916	*BRCA2, NCAPH2, PTTG1, GEM, RAD54L*
Protein heterotrimerization	5	0,002	3,463	*C1QTNF1, COL6A2, COL1A2, COL6A1, COL1A1*
Activation of protein kinase activity	7	0,003	4,734	*KIF14, CLSPN, SLC11A1, HMGB1, VEGFA, TPX2, ECT2*
CENP-A containing nucleosome assembly	4	0,003	4,822	*OIP5, HJURP, MIS18A, CENPP*
Mismatch repair	6	0,003	5,608	*EXO1, RPA1, RPA2, PCNA, RNASEH2A, RPA3*
Cell chemotaxis	10	0,004	6,411	*CCL24, HMGB2, CCL2, SAA2, CXCL3, BCAR1, SAA3, PDGFRA, PDGFRB, HGF*
Angiogenesis	19	0,004	6,663	*PRKCA, FGFR1, CAV1, EPAS1, ANG2, CCDC80, MFGE8, ESM1, MMP2, MEIS1, HOXB3, HIF1A, ANG, PLXDC1, SERPINE1, PECAM1, VEGFA, ANGPT2, FN1*
Negative regulation of extrinsic apoptotic signaling pathway via death domain receptors	6	0,004	7,440	*ICAM1, HMGB2, SERPINE1, HGF, TNFAIP3, BRCA1*
Inner cell mass cell proliferation	5	0,005	7,955	*GINS1, NCAPG2, GINS4, BRCA2, PALB2*
Locomotory behavior	12	0,005	8,386	*PAK6, APP, MCOLN3, ADORA2A, ALDH1A3, ASL, ETV5, MEIS1, ZNF385A, SNAP25, RASD2, CDH23*
Locomotory behavior	12	0,005	8,386	*PAK6, APP, MCOLN3, ADORA2A, ALDH1A3, ASL, ETV5, MEIS1, ZNF385A, SNAP25, RASD2, CDH23*
Positive regulation of mitotic cell cycle	7	0,005	9,237	*CCNB1, PRKCA, APP, BRCA2, BIRC5, ASNS, CDC25B*
Cell migration	17	0,006	9,684	*PTPRF, CD248, FSCN1, PTK7, CSPG4, WWC1, CDH2, ASAP3, PALLD, SDC4, SNAI1, ELMO3, SDC3, FAM83D, JUP, SDC1, DEPDC1B*
Osteoblast differentiation	14	0,006	10,990	*MEF2C, IFT80, FIGNL1, TNC, FHL2, GJA1, SNAI2, SNAI1, LGR4, SMO, COL6A1, COL1A1, TMEM119, SPP1*
G2 DNA damage checkpoint	6	0,007	12,193	*CLSPN, PLK1, DTL, CHEK1, BRCA1, RBBP8*
Double-strand break repair	9	0,008	13,975	*RECQL4, MSH2, TDP1, MND1, PARP3, CHEK2, PARP1, CDCA5, TRIP13*
Regulation of G2/M transition of mitotic cell cycle	4	0,009	14,495	*KIF14, CCNB3, CENPF, CCNA2*
Protein K6-linked ubiquitination	4	0,009	14,495	*UBE2S, UBE2T, BRCA1, BARD1*
Metaphase plate congression	4	0,009	14,495	*FAM83D, KIF22, CENPF, GEM*

Regulation of microtubule polymerization or depolymerization	4	0,009	14,495	*SKA3, SKA2, STMN1, SKA1*
Spindle organization	5	0,009	14,914	*CEP72, SPAG5, CKAP5, KNSTRN, ASPM*
Positive regulation of cyclin-dependent protein serine/threonine kinase activity	6	0,009	15,132	*CKS1B, CCND3, PDGFB, CCND2, PSRC1, CKS2*
Mitotic spindle assembly	7	0,010	15,960	*KIFC1, KIF11, NEK2, TPX2, BIRC5, CHEK2, MYBL2*
Protein phosphorylation	18	0,011	17,488	*IRAK2, PRKCA, NUAK2, NEK3, NUAK1, NEK2, CCL8, NPR2, BIRC5, AURKB, PBK, WNK2, CDC25B, CCNE1, PLK4, VRK1, APP, PLK1*
Regulation of cell shape	15	0,011	17,585	*CCL2, ARHGAP18, FBLIM1, ARHGAP35, ARHGAP15, CCL24, EPB41L3, MYO10, VRK1, CORO1A, CDC42EP1, VEGFA, CDC42EP4, MYH10, FN1*
Mitochondrial DNA repair	3	0,013	20,910	*DNA2, MGME1, PARP1*
Positive regulation of cell proliferation by VEGF-activated platelet derived growth factor receptor signaling pathway	3	0,013	20,910	*VEGFA, PDGFRA, PDGFRB*
Spindle checkpoint	3	0,013	20,910	*BIRC5, SPDL1, AURKB*
Centromere complex assembly	3	0,013	20,910	*CENPO, CENPN, CENPI*
Regulation of mitotic metaphase/anaphase transition	4	0,013	21,230	*CDC6, CDC23, CENPE, UBE2C*
DNA synthesis involved in DNA repair	4	0,013	21,230	*POLH, CDKN2D, POLD1, POLE*
Endothelial cell chemotaxis	4	0,013	21,230	*HMGB1, EGR3, VEGFA, RAB13*
Tetrahydrofolate interconversion	4	0,013	21,230	*MTHFD1, SHMT1, MTHFS, TYMS*
Regulation of cell cycle	11	0,015	23,996	*CCNE2, E2F2, CCNE1, ADARB1, MADD, FIGNL1, DTL, PRR11, RBL1, GRK5, MASTL*
Negative regulation of cell proliferation	24	0,021	31,336	*ADARB1, NDN, CLMN, E2F7, ITGA1, GJA1, ZNF503, WNK2, HMGA1, KANK2, TNS2, SERPINE2, CDKN2C, CDKN2D, GATA3, TSPAN32, SCIN, SFRP4, MTBP, IRF1, ROR2, C24H18ORF54, KLF4, NEURL1*
Negative regulation of neuron apoptotic process	12	0,036	48,619	*MEF2C, KIF14, NES, CORO1A, CCL2, MSH2, STXBP1, AXL, BIRC5, GDNF, CACNA1A, TP73*
Sensory perception of sound	12	0,042	53,581	*CCDC50, FGFR1, TUB, THRB, FAM107B, CDKN2D, CEMIP, COL1A1, SNAI2, CRYM, CDH23, GJB2*
Positive regulation of cell migration	15	1,262	57,703	*PRKCA, CORO1A, SYNE2, ARHGEF39, PDGFB, CXCL16, CEMIP, PDGFRA, ROR2, COL1A1, HGF, SNAI2, LAMB1, LOC520336, SNAI1*
