# Supplementary material for: Lipopolysaccharide triggers different transcriptional signatures in taurine and indicine cattle macrophages: Reactive oxygen species and potential outcomes to the development of immune response to infections
Source: PLoS One. 2020 Nov 6;15(11):e0241861. doi: 10.1371/journal.pone.0241861 (PMC7647108; doi:10.1371/journal.pone.0241861)
Supplement: S10 Table — Table showing key transcription factors (KeyTF) displaying the scores for RIF1: TF that are consistently most differentially co-expressed with the highly abundant and highly DEGs in Gir and Holstein MDMs; RIF2: TF with the most altered ability to predict the abundance of DEGs in Gir and Holstein MDMs. The frequencies for each KeyTF were calculated for Holstein and Gir MDM stimulated with LPS. The differential frequencies were also calculated for each KeyTF in order to infer their importance on MDM response to LPS treatment for each Holstein and Gir breeds. (PDF) [file pone.0241861.s012.pdf]

TF	Average Expresion	RIF1	RIF2	Frequency in Holstein_LPS	Frequency in Gir_LPS	Differential Frequency (Holstein – Gir)
E2F8	3.91553	2.37324	1.36998	29	7	22
CUX1	7.57722	-0.30703	3.25340	24	8	16
MYC	5.76217	-1.03674	2.94426	25	12	13
TSC22D1	5.98332	2.03519	0.52269	26	13	13
FLI1	6.36778	-1.01994	2.04188	26	14	12
IRF2	6.00328	0.91874	2.67065	21	10	11
ZNF142	5.50126	2.05215	0.45196	27	16	11
TAF8	4.84659	-0.27405	2.09651	18	8	10
ZC3H7A	6.39477	-2.32977	0.87170	25	15	10
GTF2I	6.09357	-0.82442	2.01626	19	10	9
MXI1	5.74478	-2.09161	0.48014	22	13	9
ZBTB18	4.41666	-2.32877	0.89658	23	14	9
BATF3	4.29897	0.51089	2.04222	21	13	8
MNT	4.49670	2.51030	1.58707	21	13	8
HBP1	5.75490	0.73749	2.84071	17	10	7
PKHD1	0.82411	-0.29128	3.56268	17	10	7
TUT1	4.94416	0.61613	3.04774	18	11	7
ID2	5.24033	-0.26402	2.23569	19	13	6
SFMBT2	4.30890	-2.22766	0.54511	21	15	6
FOSL1	4.77962	0.44143	2.43477	17	13	4
MXD1	6.27695	-2.25822	0.14805	20	16	4
SRF	5.60007	0.34542	3.29078	15	12	3
MTA2	6.55964	1.46506	2.64859	16	14	2
REPIN1	4.61493	-2.47698	-0.23097	21	19	2
TSC22D3	7.04667	1.64337	2.67970	16	14	2
EGR1	7.94059	2.28049	-0.13196	18	17	1
TEF	4.75187	-0.27136	2.07918	11	10	1
RERE	5.80801	-2.16845	-0.23530	19	19	0
ATXN7L3	6.76228	-2.15893	-0.93656	16	17	-1
RFC1	4.78622	-2.01765	-0.22436	18	19	-1
IRF5	7.88467	0.13859	2.65195	11	13	-2
SUPT20H	5.61160	-0.16587	-2.24707	16	18	-2
HMGB1	7.08521	-0.10129	-2.56997	11	15	-4
NSD2	6.15386	2.21723	-0.06884	11	16	-5
HHEX	4.76990	0.84831	-1.96002	10	18	-8
ZFHX3	5.77263	-0.89435	-1.97037	10	18	-8
ATMIN	5.34585	-0.20755	-2.17480	13	22	-9
