# Supplementary material for: Lipopolysaccharide triggers different transcriptional signatures in taurine and indicine cattle macrophages: Reactive oxygen species and potential outcomes to the development of immune response to infections
Source: PLoS One. 2020 Nov 6;15(11):e0241861. doi: 10.1371/journal.pone.0241861 (PMC7647108; doi:10.1371/journal.pone.0241861)
Supplement: S11 Table — CeTF co-expression analysis of LPS treated MDMs displayed all DEGs associated to KeyTF for each bovine breed. Overlap of co-expression networks in the Cystoscape software with Diffany plugin revealed genes found in genome wide association studies that make one unique connection to KeyTF. (PDF) [file pone.0241861.s013.pdf]

KEYTF	GENE	REFERENCES
ATMIN	*QPCT*	
	TMEM47	
ATXN7L3	*MMP25*	
BATF3	*ENSBTAG00000006383*	
	FEN1	
	MCM2	
	PROKR2	
CUX1	*ERRFI1*	
EGR1	*CREG1*	
	PTGDS	
	RNASET2	
FLI1	*SPP1*	[65-67,75,79]
FOSL1	*C1R*	[49]
	MYO10	
GTF2I	*ANKRD22*	
	ARRDC4	
	PCYOX1	
HBP1	*ARAF*	
HHEX	*H2AFZ*	
	M-SAA3.2	[70-72]
HMGB1	*TM4SF19*	
ID2	*FAM122B*	
	MPHOSPH9	
IRF2	*LOC514011*	
	SLC13A3	
IRF5	*ENSBTAG00000002290*	
MNT	*OSMR*	[69,76]
MTA2	*CDC42EP1*	
MXD1	*CD82*	[68]
MXI1	*BIRC5*	
MYC	*CITED4*	
	MMP3	
REPIN1	*IL36A*	[82]
	LOC616942	
	SLC37A2	
RERE	*DUT*	
	RACGAP1	
RFC1	*ENSBTAG00000034185*	

	ENSBTAG00000051047	
	FAM20C	
	MYL9	
SFMBT2	*ENSBTAG00000051191*	
	SERPINE1	
SRF	*CLMP*	
	CSF1	
	RCC1	
	TGM1	
SUPT20H	*ASS1*	[73, 80]
	CCDC88C	[78]
	CPT2	
	LYZ	[69]
	MYOF	[74]
	TLE5	
	VSTM1	
TEF	*LILRA4*	
TSC22D1	*ANLN*	
	ASL	[81]
	CLEC4E	[64]
	CORO7	
	ENSBTAG00000046633	
	EZH2	
	FAT1	
	NAPRT	
	PIM3	
	PTGS2	
TUT1	*TENM4*	
ZFHX3	*PAM*	
	SLC13A5	
	SLC35A1	
	USP1	
ZNF142	*ABHD6*	
	ATP2A3	[77]
	HACD4	
	PARP1	
	RAD54L	
